# Cross-Silo Federated Learning for Predicting Successful Mechanical Ventilation Weaning Across 5 Intensive Care Unit Databases: Retrospective Database Analysis

**DOI:** 10.2196/79713

**Published:** 2026-08-14

**Authors:** Seyedmostafa Sheikhalishahi, Mathias Kaspar, Johanna Schwinn, Matthaeus Morhart, Philipp Simon, Ludwig Christian Hinske

**Affiliations:** 1 Digital Medicine University Hospital Augsburg Augsburg Germany; 2 Anesthesiology and Operative Intensive Care Faculty of Medicine University of Augsburg Augsburg Germany

**Keywords:** AUMC, cross-silo federated learning, eICU-CRD, HiRID, intensive care unit, machine learning, mechanical ventilation, MIMIC-IV, UKA, weaning

## Abstract

**Background:**

The prediction of weaning from mechanical ventilation (MV) can support clinical decision-making and help reduce the risk of weaning failure in intensive care units (ICUs). Cross-silo federated learning (FL) offers a promising approach to developing robust predictive models across multiple institutions without requiring the sharing of patient-level data.

**Objective:**

This study aimed to evaluate the feasibility and efficacy of FL for predicting successful weaning from MV across 5 diverse ICU databases and to compare its performance with local learning (LL) and centralized learning (CL) approaches that differ in their data-sharing requirements.

**Methods:**

We conducted a retrospective analysis using 5 ICU databases, namely the eICU Collaborative Research Database (eICU-CRD), Medical Information Mart for Intensive Care IV (MIMIC-IV), Universitätsklinikum Augsburg (UKA), High-Resolution ICU Dataset (HiRID), and Amsterdam University Medical Centers (AUMC), transforming clinical variables into the Observational Medical Outcomes Partnership (OMOP) Common Data Model. We defined successful weaning as a sustained reduction in positive end-expiratory pressure. We compared 3 learning approaches, FL, LL, and CL, using extreme gradient boosting (XGBoost). Performance was evaluated using the area under the receiver operating characteristic curve (AUROC), area under the precision-recall curve (AUPRC), precision, recall, and *F*_1_-score. All data use complied with local ethical regulations and institutional review board approvals, and all databases contained deidentified patient data accessed under institutional data use agreements and governed by applicable privacy regulations.

**Results:**

A total of 24,521 patients were included across 5 databases. The CL model achieved an AUROC of 0.81, AUPRC of 0.57, and *F*_1_-score of 0.54 on pooled test data. The FL model achieved a macroaveraged AUROC of 0.74, AUPRC of 0.56, and *F*_1_-score of 0.52. LL model performance varied across databases (AUROC=0.68-0.84, AUPRC=0.51-0.68, *F*_1_-score=0.49-0.67), reflecting differences in data distribution and class balance.

**Conclusions:**

Our findings highlight performance differences between learning approaches for MV weaning prediction. LL models achieved the highest performance within their respective institutions (AUROC=0.68-0.84), CL achieved the highest performance on pooled test data (AUROC=0.81), and FL showed lower but reasonable performance while avoiding direct sharing of patient-level data (AUROC=0.74). The choice between approaches depends on institutional data-sharing constraints, local dataset characteristics, and acceptable performance thresholds. As privacy was not formally measured, these findings should be read as a performance comparison rather than a privacy-performance trade-off.

## Introduction

Mechanical ventilation (MV) is a critical life-support intervention required by 50% to 70% of patients in intensive care units (ICUs) globally [1-5]. Although the necessity of MV is clear, the process of liberating patients from ventilator support, known as weaning, represents one of the most challenging decisions in critical care medicine [6,7]. The complexity of this decision is heightened by the fact that both premature and delayed weaning attempts can lead to adverse outcomes, including prolonged ICU stays and increased mortality [7,8].

Positive end-expiratory pressure (PEEP) serves as a fundamental parameter in MV, playing multiple crucial roles: enhancing oxygenation, preventing alveolar collapse, and optimizing ventilation-perfusion matching [9-11]. PEEP reduction serves as a valuable predictor for weaning readiness, as it reflects several aspects of respiratory recovery. As patients improve clinically, decreasing PEEP levels indicate enhanced lung function, with lower PEEP requirements suggesting improved oxygenation capacity, reduced alveolar recruitment dependency, and better respiratory mechanics. In clinical practice, successful liberation from ventilator support is typically preceded by stepwise PEEP reductions, making this parameter particularly suitable as a predictive target for weaning success [12].

Recent advances in machine learning have led to various approaches for predicting weaning outcomes [13-16], including applications of extreme gradient boosting (XGBoost), convolutional neural networks, and interpretable machine learning models that have shown promise in intensive care settings. XGBoost has emerged as a powerful algorithm in clinical prediction tasks due to its ability to handle complex, multivariate data [17]. However, developing robust prediction models in health care faces a fundamental challenge: while large-scale, diverse databases are crucial for model accuracy, health care data are highly sensitive and typically sit in institutional silos with strict privacy regulations limiting data sharing [18]. Traditional approaches requiring centralized data pools [19] often fail to fully exploit existing medical data due to these privacy constraints and governance challenges. In this context, multicenter ICU databases, which compile data from different institutions into one centralized repository, have been used for benchmarking machine learning models in critical care settings, demonstrating the feasibility of developing robust predictive models across diverse patient populations [19]. While this centralized data pooling approach has proven valuable, it still highlights the importance of leveraging data from multiple institutions without requiring direct data sharing, a key motivation for federated learning (FL) in clinical applications.

FL has emerged as a promising approach for collaborative model development by keeping patient-level data within institutional boundaries and sharing only model updates or artifacts. While this architectural property is often described as privacy-enhancing, it is important to note that shared model artifacts (eg, gradients or tree structures) may still encode information about the underlying training data, and the actual privacy properties of any specific FL implementation depend on the aggregation strategy and any additional defenses applied [20]. In this framework, a central aggregation server orchestrates the training process through a Hub and Spoke model, where the server coordinates training iterations and facilitates model distribution and parameter aggregation across participating institutions. Each institution trains the model locally on its private data, sharing only model updates with the central server. For neural networks, these updates are then aggregated using techniques such as federated averaging (FedAvg) [21], while tree-based models use ensemble-based strategies such as bagging, where locally grown trees are combined into a global model. The resulting global model is then redistributed to all participants for further refinement [22].

This FL approach has shown promising results across various health care applications. Studies have demonstrated that FL-trained models can achieve performance levels comparable to those trained on centrally hosted databases and superior to models trained only on isolated single-institutional data [23]. Recent research has shown the feasibility of FL across a range of clinical tasks, from brain tumor segmentation [24] to ICU mortality prediction [22,25]. In some of these studies, federated models have achieved performance comparable to centralized learning (CL) baselines, though the extent of this parity varies. Complementary work has also evaluated different FL strategies against each other; for example, personalized FL via patient clustering has shown benefits in heterogeneous cohorts [26], indicating that methodological choices within the FL paradigm (aggregation strategy and personalization) are themselves important drivers of performance.

Specialized ICU-focused federated frameworks, such as FedICU, have been developed for clinical decision support tasks, including medication-error reduction in ICUs, applying Paillier homomorphic encryption and differential privacy to protect model updates [27]. The FedICU work also explicitly quantifies a privacy-performance trade-off under differential privacy, illustrating that formal privacy measurement is feasible in federated ICU applications. Additionally, FL has proven particularly effective for rare disease applications, as demonstrated by the Federated Tumor Segmentation initiative involving 71 international sites, which reported a 33% improvement in delineating the surgically targetable tumor and a 23% improvement for the complete tumor extent compared with a model trained on publicly available data alone [24]. This makes FL particularly valuable for clinical tasks such as ventilator weaning prediction, where access to diverse patient populations and clinical scenarios is crucial for model robustness and generalizability, but data sharing is restricted by privacy regulations and institutional policies.

Despite the promise of FL in health care, critical questions remain about its practical implementation across heterogeneous ICU databases with varying data quality, documentation practices, and patient populations. This study evaluates FL for predicting successful weaning from MV across 5 diverse ICU databases (eICU Collaborative Research Database [eICU-CRD], Medical Information Mart for Intensive Care IV [MIMIC-IV], Universitätsklinikum Augsburg [UKA], High-Resolution ICU Dataset [HiRID], and Amsterdam University Medical Centers [AUMC]), comparing its performance to local learning (LL) and CL approaches. We further assess how data heterogeneity (variations in data quality, class distribution, and documentation practices) affects model performance across these 3 approaches, which differ in their data-sharing requirements. Our federated setup avoids sharing of patient-level data by design, but privacy was not formally measured in this study.

## Methods

### Study Design

This is a retrospective study based on 5 databases containing data from different ICUs: eICU-CRD [28], MIMIC-IV v2.2 [29], UKA, HiRID [30], and AUMC [31]. The objective is to predict a successful reduction in PEEP as part of the weaning process from MV. We define the outcome, which serves as the prediction target, as a reduction in the PEEP value that is not followed by any subsequent increase during the ICU stay. If PEEP is reduced and remains stable or continues to decrease, the outcome is labeled as a positive outcome (successful). If the PEEP value increases again after the initial reduction, it is labeled as a negative outcome (unsuccessful).

Outcome assessment followed each patient through their ICU stay, with positive outcomes defined as sustained PEEP reduction without subsequent increase. Patients were censored at ICU discharge or transfer; PEEP reductions occurring without sufficient subsequent observation (eg, due to early discharge or transfer) were classified based on the last available PEEP value before censoring. Deaths occurring within 48 hours of the last observation were classified as unsuccessful weaning attempts, preventing misclassification of terminal extubation as successful weaning. Deaths occurring beyond 48 hours after a sustained PEEP reduction were not reclassified, as the weaning itself was considered clinically successful.

To ensure consistency across the different data sources, all clinical variables from the 5 databases were transformed into the Observational Medical Outcomes Partnership (OMOP) Common Data Model (CDM) [32]. The OMOP CDM standardizes clinical concepts using consistent vocabularies (Logical Observation Identifiers Names and Codes [LOINC] for laboratory tests and Systematized Nomenclature of Medicine-Clinical Terms [SNOMED-CT] for clinical findings) and units of measurement (Unified Code for Units of Measure). Our OMOP mapping involved: (1) mapping source concepts to standard OMOP concept IDs, (2) converting measurements to standardized units (eg, temperature to Celsius and blood pressure to mm Hg), (3) temporal alignment relative to PEEP reduction events, and (4) quality control validation of semantic consistency across sites. We extracted the same 39 clinical variables from all databases and applied uniform temporal processing (24-hour observation windows and standardized padding/truncation). However, differences in measurement frequency and missingness patterns mean that the same variable may have different temporal resolution and completeness across sites, potentially affecting feature importance patterns in unpredictable ways.

The data were preprocessed and used to train machine learning models after data harmonization. These models were trained and evaluated in different configurations: FL, where models are trained locally on each database and aggregated on the server without sharing patient data; LL, where models are trained on each database individually; and a CL model, where models are trained on the pooled data when permissible.

### Data Sources

We used 5 critical care databases from different institutions and countries. A summary of these data sources is provided in Table 1.

**Table 1:** Summary of the critical care databases used in this study.

Database	ICU^a^ stays	Time span	Country	Institution	Description
eICU-CRD^b^ [28]	200,859	2014-2015	United States	Multicenter	Data from 208 hospitals
MIMIC-IV^c^ v2.2 [29]	53,150	2008-2019	United States	Beth Israel Deaconess Medical Center	Detailed clinical data from a large hospital
UKA^d^	20,000	2010-2023	Germany	Universitätsklinikum Augsburg	Data from a German university hospital
HiRID^e^ [30]	33,000	2008-2016	Switzerland	Bern University Hospital	High-resolution physiological ICU data
AUMC^f^ [31]	23,000	2003-2016	Netherlands	Amsterdam University Medical Centers	Data from a major Dutch academic center

^a^ICU: intensive care unit.

^b^eICU-CRD: eICU Collaborative Research Database.

^c^MIMIC-IV: Medical Information Mart for Intensive Care IV.

^d^UKA: Universitätsklinikum Augsburg.

^e^HiRID: High-Resolution ICU Dataset.

^f^AUMC: Amsterdam University Medical Centers.

### Data Preprocessing

We implemented a multistage pipeline to identify successful weaning attempts from MV in terms of a successful PEEP reduction. The first stage involved a comprehensive labeling algorithm that identified clinically meaningful decreases in PEEP while filtering out transient fluctuations. We further incorporated extubation outcome validation, where successful extubations were defined by 2 criteria: absence of reintubation for at least 48 hours or patient survival beyond 48 hours after the last observation. This validation step was essential to distinguish between temporary PEEP reductions and those that led to sustainable liberation from MV, ensuring our training data captured clinically meaningful weaning attempts rather than premature or unsuccessful trials.

The feature set was constructed from 39 clinical variables, including vital signs, ventilator parameters, laboratory values, and neurological assessments. These 39 clinical variables were selected based on literature review and their clinical relevance to respiratory function, hemodynamic stability, metabolic state, and neurological status, which are the factors known to influence or reflect the need for ventilatory support adjustments. The selection was informed by prior literature [7,12,33], clinical expertise, and data availability across participating institutions to ensure consistent and meaningful input features for predictive modeling. The complete list of input features, with their distributions by outcome at each site, is provided in Tables S1-S5 in Multimedia Appendix 1. All 39 variables were used as model inputs across all sites with identical feature definitions; no site-specific feature selection was applied. Age, gender, and BMI are reported as cohort characteristics but were not included as model inputs. This was a deliberate design choice to keep the model focused on the time-varying physiological state preceding weaning, treating demographics as cohort descriptors rather than predictors. All variables were time-anchored relative to the PEEP reduction event, with each temporal sequence representing the 24 hours immediately preceding the event.

We extracted temporal sequences of these variables from each patient episode, standardizing them to 24-hour windows preceding PEEP reduction outcomes. Shorter sequences were padded with null values, while longer sequences were truncated to the most recent 24 hours. Each episode was represented as a matrix of 39 variables × 24 hourly time steps, which was flattened into a single feature vector of 936 values for input to XGBoost. The flattening preserves temporal ordering, with features indexed as variable time steps (eg, heart rate_t1 through heart rate_t24). Descriptive statistics for each of the 39 variables stratified by site and outcome are provided in Tables S1-S5 in Multimedia Appendix 1.

Data quality was ensured by filtering segments where less than 30% of values were available. We maintained the structure of missing data (null values) without imputation for the remaining missing values, leveraging XGBoost’s native capability to handle missing values efficiently during training and inference. This approach preserves the information that certain values were unavailable at specific time points, which itself may have clinical significance in the intensive care setting, while avoiding potential bias introduction through imputation. The final preprocessed dataset consisted of the flattened 24-hour × 39-variable temporal sequences for each PEEP reduction episode, paired with the binary outcome label derived from whether the PEEP reduction was successful (positive) or failed (negative) and validated against subsequent extubation outcomes.

We performed data quality assessments across all participating sites to characterize heterogeneity in documentation practices and data completeness (Figures S7-S9 in Multimedia Appendix 1).

Our analysis was done at the PEEP reduction episode level, where each episode represents a 24-hour observation window preceding a PEEP reduction event. Individual patients could contribute multiple episodes across their ICU stay, resulting in more episodes than unique patients (24,521 unique patients contributing 223,277 PEEP reduction episodes after completeness filtering across all sites). To prevent data leakage, we performed patient-level splitting: patients were randomly divided into training, validation, and testing sets (with stratification based on whether they had at least one successful weaning episode), and all episodes from a given patient were assigned to the same split. This ensures the model is evaluated on patients it has never encountered during training, providing a realistic assessment of performance for new patients.

We applied a 30% completeness threshold at the episode level, excluding sequences where less than 30% of feature–time point combinations contained values. This threshold balanced data quality requirements with sample size retention. Completeness distributions varied substantially across sites, as shown in Figure S7 in Multimedia Appendix 1, with HiRID showing the most concentrated distribution (45%-55% completeness) due to high-frequency automated monitoring, while eICU-CRD showed a bimodal distribution (10%-50% completeness) reflecting its multicenter heterogeneity across 208 hospitals.

The 30% completeness threshold had disproportionate effects across institutions, with exclusion rates ranging from 6.7% (4219/63,151; AUMC) to 86.7% (826,392/952,931; HiRID). This variation reflects fundamental differences in documentation practices: HiRID's automated high-frequency monitoring (2- to 5-minute intervals) generated a very large pool of candidate episodes (952,931), of which only 13.3% (126,539/952,931) met the completeness threshold whereas eICU-CRD's multicenter heterogeneity (208 hospitals) led to 73.5% (17,466/23,775) retention. After filtering, the final dataset comprised 223,277 episodes, with HiRID contributing 126,539 (56.7%) episodes, creating an implicit weighting toward high-frequency monitoring environments. This disproportionate filtering introduces potential selection bias, as retained episodes may systematically differ in patient acuity, clinical protocols, and resource availability. However, we maintained a uniform threshold across all sites to ensure transparency and comparability, acknowledging this limitation in our interpretation of results.

As shown in Figure S8 in Multimedia Appendix 1, feature-level missingness ranged from 10% to 100%, depending on the variable and site. Laboratory values showed the highest missingness and greatest intersite variation (albumin: 42%-99% missing; bilirubin: 15%-97% missing), while vital signs were more consistently documented but still varied substantially (heart rate: 24%-92% missing). Ventilator parameters (PEEP, peak inspiratory pressure, and fraction of inspired oxygen) showed paradoxically high missingness despite being central to the study, likely reflecting institutional practices of documenting values primarily at setting changes rather than continuously. Temporal analysis in Figure S9 in Multimedia Appendix 1 revealed that missingness increased as episodes approached the PEEP reduction event across all sites, ranging from 50%-65% at 24 hours before the event to 70%-92% at the event time, reflecting clinical workflow dynamics where documentation focus shifts toward intervention implementation as critical decisions approach.

These documentation practice differences may introduce systematic biases: automated high-frequency monitoring (HiRID) captures different clinical patterns than selective manual charting (eICU-CRD and MIMIC-IV), potentially affecting which patient states and clinical trajectories are well-represented in the training data.

### Model Development

**Figure 1:**
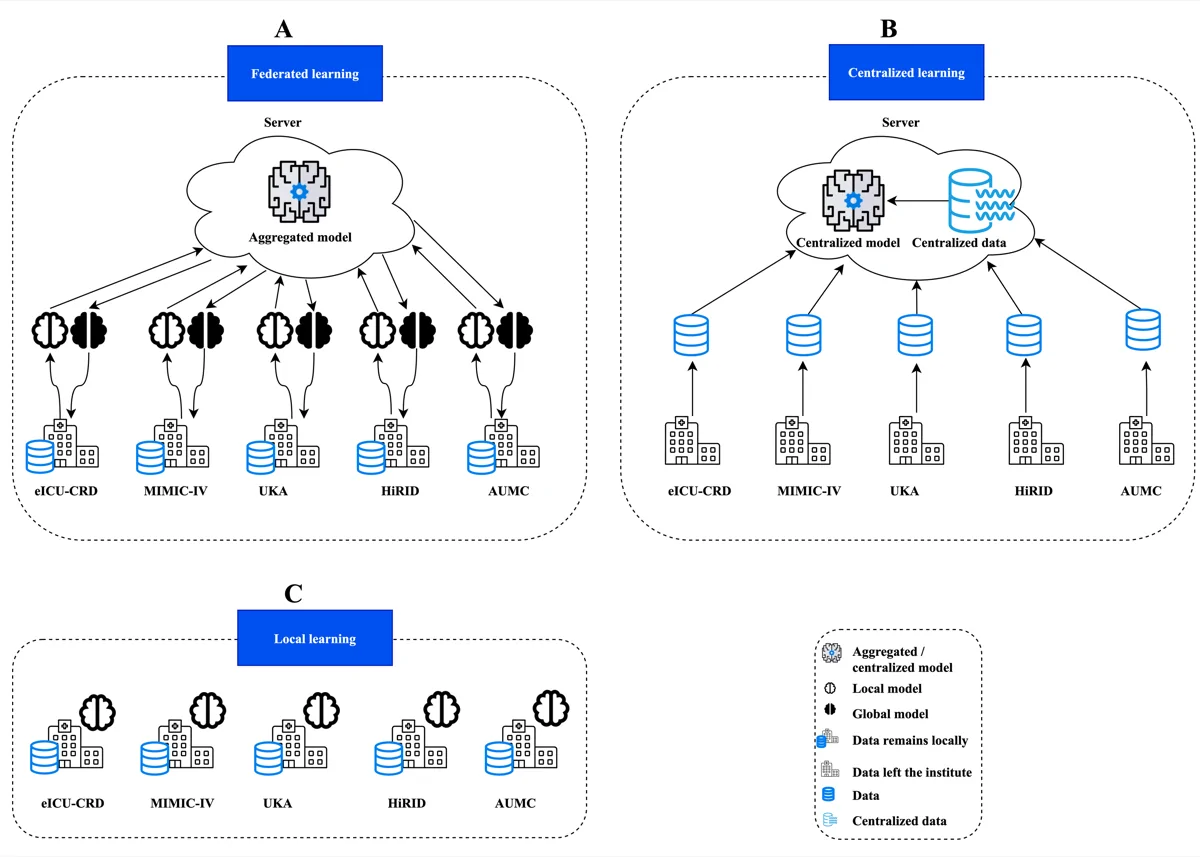
Conceptual framework of the 3 learning approaches evaluated in this study. The diagram illustrates data flow and model training architectures for (A) local learning, (B) centralized learning, and (C) federated learning. AUMC: Amsterdam University Medical Centers; eICU-CRD: eICU Collaborative Research Database; HiRID: High-Resolution ICU Dataset; MIMIC-IV: Medical Information Mart for Intensive Care IV; UKA: Universitätsklinikum Augsburg.

Figure 1 presents a conceptual overview of the 3 learning approaches. Models were trained in 3 configurations: FL, CL, and LL. In FL, data remain within their institutions, and only model parameters are shared across institutions. We implemented these approaches as a laboratory setup on a single compute node (Ubuntu 22.04.5 LTS, equipped with an Intel Xeon Gold 5215 CPU at 2.50 GHz, 250 GiB of RAM, and 2 NVIDIA RTX A6000 GPUs with 48 GiB of memory each) on various Docker containers.

Hyperparameters for the XGBoost model were selected through automated hyperparameter optimization using Optuna, focusing on balanced performance across all learning approaches (CL, LL, and FL) rather than overfitting to any single approach. Initial hyperparameter values were based on recommendations in the FL literature [34]. We conducted a series of iterative trials using Optuna, systematically varying key parameters including learning rate (range 0.01-0.3), maximum tree depth (range 3-10), number of parallel trees, and regularization parameters. The optimization focused primarily on balancing model performance metrics (area under the receiver operating characteristic curve [AUROC] and area under the precision-recall curve [AUPRC]) while maintaining a fair comparison between learning paradigms. For FL specifically, we tuned additional federation-specific parameters such as the number of global federated rounds and local epochs to optimize the convergence of the global model [34]. This balanced approach to hyperparameter optimization ensured fair evaluation of each learning paradigm without biasing model performance toward any training methodology. In LL, each institution trains and evaluates its model independently using only its local data. In CL, all data from all institutions is collected centrally for model training and evaluation. The LL and CL models were trained on single Docker containers.

For the FL model, we implemented a federated XGBoost architecture using the Flower framework (v1.8) with the federated XGBoost bagging strategy (FedXgbBagging), running on XGBoost and Python 3.10. The system consisted of 5 Docker containers running Flower clients and 1 container running the Flower server. In each federated round, each client performs 1 local boosting round on its private data and sends the resulting serialized XGBoost model (in JSON format containing all tree structures) to the server. The server aggregates models using bagging: rather than averaging parameters as in FedAvg, FedXgbBagging concatenates the trees contributed by each client into a combined ensemble model, which is then redistributed for further local boosting in the next round. This approach aligns with federated ensemble methods for tree-based models, where the global model is an ensemble of locally grown trees rather than a single model with averaged parameters. Each boosting round constructs 9 parallel trees, adding random forest–like diversity within each round.

A significant challenge was addressing class imbalance variation across institutions (18%-46% positive cases). We addressed this through dynamic weight adjustment, where each client’s model automatically adapts to its local class distribution by scaling the gradient for positive examples proportional to the local class ratio. This training-time adjustment maintains model compatibility during the federation process; evaluation was performed at a fixed 0.5 classification threshold across all sites.

### Evaluation

Continuous variables are summarized using the mean with SD, while the categorical variables are summarized using absolute and relative frequencies. Baseline characteristics of patients were compared between the target groups using the Wilcoxon-Mann-Whitney test for continuous variables and the chi-square test for categorical variables.

Models were evaluated in different scenarios with a patient-level train:validation:test data ratio of 70%:10%:20%, respectively (random state for reproducibility). Patient-level splitting ensured no patient appeared in multiple splits, eliminating data leakage, though individual patients contributed multiple episodes to their assigned split. As shown in Figure 1, federated models were evaluated on each local test set after performing local boosting. Locally trained models were evaluated on their local test sets. The centrally trained model was evaluated on the pooled test set (all institutions combined) and additionally on each institution’s local test set individually.

Model performance was assessed using the following performance metrics: AUROC, AUPRC, precision, recall, and *F*_1_-score. We included AUPRC alongside AUROC due to the substantial class imbalance observed across our datasets, as AUROC can be overly optimistic in imbalanced settings while AUPRC more accurately captures model performance when positive classes are rare. This is particularly relevant in our study, where positive class distributions varied widely (18%-46%) across institutions, making AUPRC a more clinically meaningful evaluation metric for databases with lower rates of successful weaning. A fixed classification threshold of 0.5 was applied uniformly across all approaches and sites. Performance metrics are reported in Results section, and the test set sample sizes, positive class prevalence, and detailed confusion matrix counts are provided in Table S6 in Multimedia Appendix 1.

### Ethical Considerations

This study was conducted in accordance with institutional ethical guidelines. For the UKA dataset, ethical approval was obtained from the responsible Ethics Committee at the Ludwig-Maximilians-Universität München (reference 23-0969; January 5, 2024). The eICU-CRD and MIMIC-IV databases are publicly available, deidentified datasets accessible through PhysioNet credentialed access following completion of required training courses. The HiRID and AUMC datasets were accessed under existing institutional agreements for secondary data analysis. All datasets contained deidentified patient data governed by applicable privacy regulations and institutional data use agreements. No additional patient consent was required as all data were deidentified prior to our analysis.

## Results

### Overview

A total of 24,521 patients were included in the study, with 3347 from eICU-CRD, 3494 from MIMIC-IV, 1783 from UKA, 10,040 from HiRID, and 5857 from AUMC. These patients contributed a total of 223,277 PEEP reduction episodes (Tables 2 and 3 report characteristics at the episode level, where individual patients may contribute multiple episodes).

**Table 2:** Descriptive cohort characteristics and outcomes by database^a^.

Database	Age (years), mean (SD)	BMI (kg/m^2^), mean (SD)	ICU^b^ LoS^c^ (hours), mean (SD)	MV^d^ duration (hours), mean (SD)	Gender (female), n/N (%)	Mortality, n/N (%)	Episodes retained, n
eICU-CRD^e^ (n=3347 patients)	60.9 (14.5)	29.4 (7.6)	460.2 (596.5)	416.2 (533.7)	9167/23,775 (38.6)	4083/23,775 (17.2)	17,466
MIMIC-IV^f^ (n=3494 patients)	61.4 (14.7)	32.8 (7.3)	265.4 (214.3)	130.5 (114.8)	4922/12,595 (39.1)	4535/12,595 (36.0)	11,367
UKA^g^ (n=1783 patients)	63.2 (13.0)	29.2 (5.6)	324.0 (372.9)	195.0 (218.1)	2731/10,032 (27.2)	3917/10,032 (39.0)	8973
HiRID^h^ (n=10,040 patients)	61.1 (14.8)	26.7 (4.3)	369.5 (204.4)	121.6 (70.6)	292,929/952,931 (30.7)	89,081/952,931 (9.3)	126,539
AUMC^i^ (n=5857 patients)	60.8 (15.5)	26.5 (4.2)	604.1 (536.4)	356.0 (298.6)	21,656/63,151 (34.3)	32,524/63,151 (51.5)	58,932

^a^All variables are summarized at the episode level. Gender and mortality are reported as n/N (%), where N is the number of labeled positive end-expiratory pressure reduction episodes in that database before the 30% completeness filter; individual patients may contribute multiple episodes. The patient count in each row header is the number of patients contributing at least one episode retained after the filter. “Episodes retained” is the number of episodes remaining after the filter and is therefore smaller than N.

^b^ICU: intensive care unit.

^c^LoS: length of stay.

^d^MV: mechanical ventilation.

^e^eICU-CRD: eICU Collaborative Research Database.

^f^MIMIC-IV: Medical Information Mart for Intensive Care IV.

^g^UKA: Universitätsklinikum Augsburg.

^h^HiRID: High-Resolution ICU Dataset.

^i^AUMC: Amsterdam University Medical Centers.

**Table 3:** Descriptive cohort characteristics stratified by outcome across 5 intensive care databases^a^.

Database			Age (years), mean (SD)		BMI (kg/m^2^), mean (SD)		ICU^b^ LoS^c^ (hours), mean (SD)		MV duration (hours), mean (SD)		Gender (female), n/N (%)		Mortality, n/N (%)
**eICU-CRD^d^**													
	Positive	60.5 (15.0)		30.2 (7.5)		242.3 (267.0)		219.4 (255.3)		3031/7903 (38.4)		1055/7903 (13.3)	
	Negative	61.1 (14.3)		29.0 (7.6)		568.7 (679.8)		513.2 (603.8)		6136/15,872 (38.7)		3028/15,872 (19.1)	
	*P* value	.10		<.001		<.001		<.001		.66		<.001	
**MIMIC-IV^e^**													
	Positive	60.6 (14.6)		33.2 (7.3)		211.6 (183.0)		110.0 (103.6)		2103/5824 (36.1)		1672/5824 (28.7)	
	Negative	62.2 (14.8)		32.4 (7.2)		311.7 (228.0)		148.2 (120.8)		2819/6771 (41.6)		2863/6771 (42.3)	
	*P* value	<.001		<.001		<.001		<.001		<.001		<.001	
**UKA^f^**													
	Positive	63.6 (13.0)		29.2 (5.6)		252.2 (278.6)		146.1 (167.8)		1154/4008 (28.8)		1355/4008 (33.8)	
	Negative	62.9 (13.0)		29.2 (5.7)		371.6 (417.4)		231.4 (242.6)		1577/6024 (26.2)		2562/6024 (42.5)	
	*P* value	.005		.73		<.001		<.001		.004		<.001	
**HiRID^g^**													
	Positive	62.5 (14.5)		26.8 (4.3)		240.6 (203.2)		84.5 (68.6)		31,709/108,545 (29.2)		7551/108,545 (7.0)	
	Negative	60.9 (14.9)		26.7 (4.3)		386.1 (198.5)		126.3 (69.5)		261,220/844,386 (30.9)		81,530/844,386 (9.7)	
	*P* value	<.001		<.001		<.001		<.001		<.001		<.001	
**AUMC^h^**													
	Positive	60.8 (15.5)		26.5 (4.1)		312.7 (345.1)		189.6 (206.7)		4158/12,857 (32.3)		5144/12,857 (40.0)	
	Negative	60.8 (15.5)		26.5 (4.2)		678.6 (550.9)		398.5 (303.6)		17,498/50,294 (34.8)		27,380/50,294 (54.4)	
	*P* value	.95		.02		<.001		<.001		<.001		<.001	

^a^All variables are summarized at the episode level. Gender and mortality are reported as n/N (%), where N is the number of episodes in that outcome group. *P* values are from Mann-Whitney *U* tests for continuous variables and chi-square tests for categorical variables, computed at the episode level.

^b^ICU: intensive care unit.

^c^LoS: length of stay.

^d^eICU-CRD: eICU Collaborative Research Database.

^e^MIMIC-IV: Medical Information Mart for Intensive Care IV.

^f^UKA: Universitätsklinikum Augsburg.

^g^HiRID: High-Resolution ICU Dataset.

^h^AUMC: Amsterdam University Medical Centers.

### Data Quality Across Sites

Substantial variations in data quality and documentation practices were observed across the 5 institutions. Episode-level completeness distributions showed distinct institutional patterns: HiRID demonstrated the highest data quality with 45% to 55% completeness concentrated in a narrow range, reflecting its high-resolution monitoring system (2- to 5-minute intervals for vital signs). In contrast, eICU-CRD showed a bimodal distribution with peaks near 10% and 50%, reflecting documentation heterogeneity across its 208 contributing hospitals.

Feature-specific missingness patterns showed systematic differences in institutional practices. Laboratory values showed the greatest variation, with albumin missingness ranging from 42% (MIMIC-IV) to 99% (eICU-CRD). Vital signs demonstrated better overall documentation but still varied substantially across sites, with heart rate missingness ranging from 24% to 92%. Ventilator parameters showed paradoxically high missingness despite being central to the study, likely reflecting documentation practices that record values primarily at changes rather than continuously.

Temporal missingness analysis showed a consistent pattern across all institutions: data completeness decreased as episodes approached the PEEP reduction event, with missingness increasing from approximately 50% to 65% at 24 hours before the event to 70% to 92% at the event time. This pattern suggests a clinical focus shifts toward intervention rather than routine documentation as critical decisions approach. Despite these variations, all sites maintained sufficient data quality above our 30% completeness threshold to enable meaningful model training. After applying the 30% threshold, retention rates varied from 13.3% (n=126,539, HiRID) to 93.3% (n=58,932, AUMC), with HiRID contributing 56.7% (n=126,539) of the final 223,277 episodes.

### Patient Characteristics

Table 2 shows basic patient characteristics of the 5 used databases, showing substantial variations. The mean age was relatively consistent, ranging from 60.9 years in eICU-CRD to 63.2 years in UKA. Gender distribution varied, with female representation highest in MIMIC-IV (4922/12,595, 39.1%) and lowest in UKA (2731/10,032, 27.2%). Clinical outcomes showed substantial heterogeneity; the length of stay in ICU showed substantial variation, with AUMC reporting the longest ICU length of stay (mean 604.1, SD 536.4 hours) and MIMIC-IV the shortest (mean 265.4, SD 214.3 hours). MV duration also varied considerably, with eICU-CRD patients requiring the longest ventilation support (mean 416.2, SD 533.7 hours) and HiRID patients the shortest (mean 121.6, SD 70.6 hours). Mortality rates differed dramatically across institutions, with AUMC reporting the highest mortality (32,524/63,151, 51.5%) and HiRID the lowest (89,081/952,931, 9.3%). Correlation matrices of all clinical variables across the 5 databases are shown in Figures S1-S5 in Multimedia Appendix 1, and the correlation matrix of the combined dataset is shown in Figure S6 in Multimedia Appendix 1.

Moreover, Table 3 shows the patient characteristics of each database in which single outcomes are counted separately. Patients in the positive outcome group generally had shorter ICU stays, MV durations, and lower mortality across all 5 databases. Significant differences were also observed in age, gender, and BMI. There are significant variations in initial outcome distribution among databases.

A detailed overview of all clinical variables in the analysis by database is provided in Tables S1-S5 in Multimedia Appendix 1. Several consistent patterns were shown in Tables S1-S5 in Multimedia Appendix 1: patients with positive outcomes typically had higher oxygen partial pressure (*P*<.001 across all databases), better oxygenation capacity, and lower hemodynamic parameters. Laboratory values, including hemoglobin and albumin, were consistently higher in the positive outcome group, while metabolic markers such as urea nitrogen were lower.

The observed heterogeneity across these 5 databases in patient demographics, clinical variables, and outcome distributions creates an ideal environment for evaluating different learning approaches. In the following sections, we present performance comparisons of 3 distinct learning approaches: FL, CL, and LL models trained on individual databases.

### Comparative Performance of Learning Approaches

**Figure 2:**
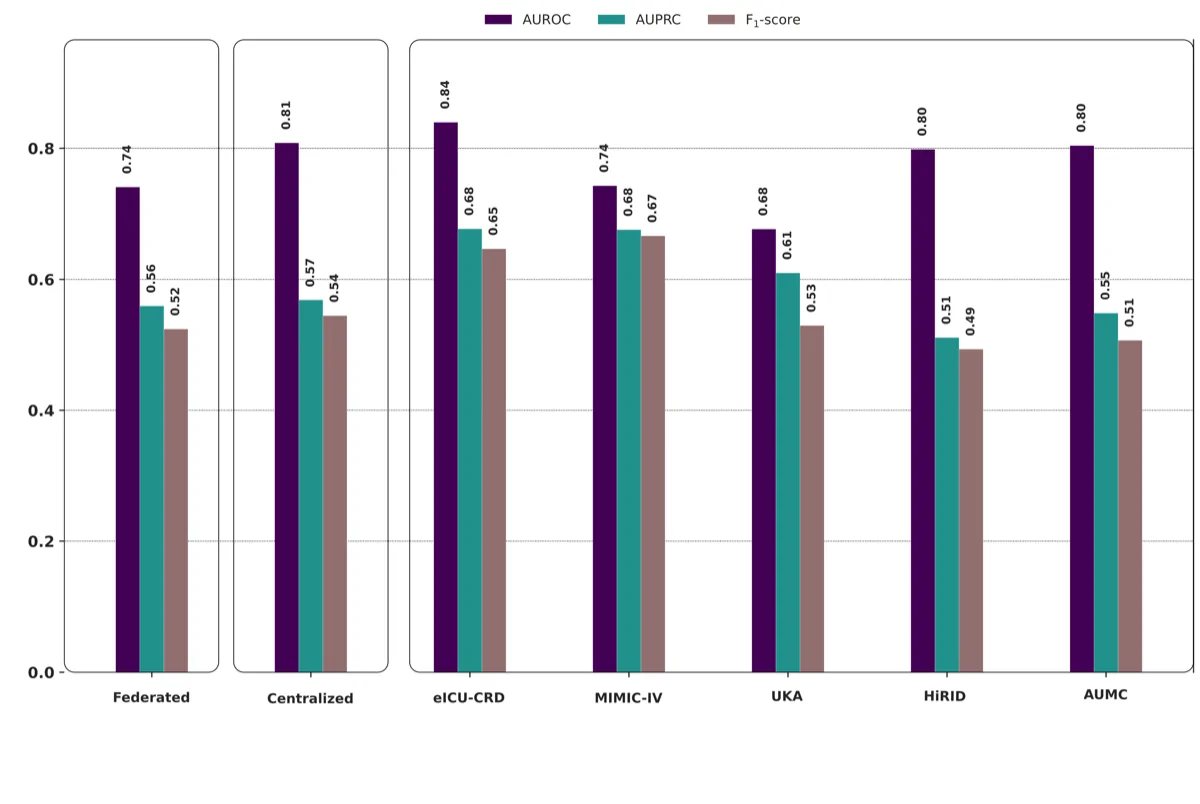
Comparison of performance metrics across 3 training approaches: federated learning, centralized learning, and local learning for each database, showing the relative strengths of each approach. AUMC: Amsterdam University Medical Centers; AUPRC: area under the precision-recall curve; AUROC: area under the receiver operating characteristic curve; eICU-CRD: eICU Collaborative Research Database; HiRID: High-Resolution ICU Dataset; MIMIC-IV: Medical Information Mart for Intensive Care IV; UKA: Universitätsklinikum Augsburg.

We evaluated 3 learning approaches: FL, CL, and LL across 5 ICU databases to predict successful MV weaning. As shown in Table 4 and Figure 2, the CL model achieved an AUROC of 0.81, AUPRC of 0.57, precision of 0.47, recall of 0.65, and *F*_1_-score of 0.54 on pooled test data. The FL approach achieved a macroaveraged AUROC of 0.74, AUPRC of 0.56, precision of 0.55, recall of 0.51, and *F*_1_-score of 0.52. LL performance varied considerably across institutions; eICU-CRD and MIMIC-IV LL models achieved the highest *F*_1_-scores (0.65 and 0.67, respectively), coinciding with their more balanced class distributions of approximately 35% (6169/17,466) and 46% (5196/11,367) positive cases, respectively. In contrast, HiRID and AUMC models, with highly imbalanced distributions (22,471/126,539, 18% and 11,579/58,932, 20% positive cases, respectively), demonstrated lower precision and *F*_1_-scores despite achieving reasonable AUROC values.

**Table 4:** Performance comparison of federated, centralized, and local learning approaches.

Learning approach			AUROC^a^		AUPRC^b^		Precision		Recall	*F*_1_-score
Centralized			0.81		0.57		0.47		0.65	0.54
Federated (macro average)			0.74		0.56		0.55		0.51	0.52
**Local**										
	eICU-CRD^c^	0.84		0.68		0.57		0.75		0.65
	MIMIC-IV^d^	0.74		0.68		0.62		0.72		0.67
	UKA^e^	0.68		0.61		0.58		0.49		0.53
	HiRID^f^	0.80		0.51		0.42		0.61		0.49
	AUMC^g^	0.80		0.55		0.48		0.54		0.51

^a^AUROC: area under the receiver operating characteristic curve.

^b^AUPRC: area under the precision-recall curve.

^c^eICU-CRD: eICU Collaborative Research Database.

^d^MIMIC-IV: Medical Information Mart for Intensive Care IV.

^e^UKA: Universitätsklinikum Augsburg.

^f^HiRID: High-Resolution ICU Dataset.

^g^AUMC: Amsterdam University Medical Centers.

### FL vs LL Performance

**Figure 3:**
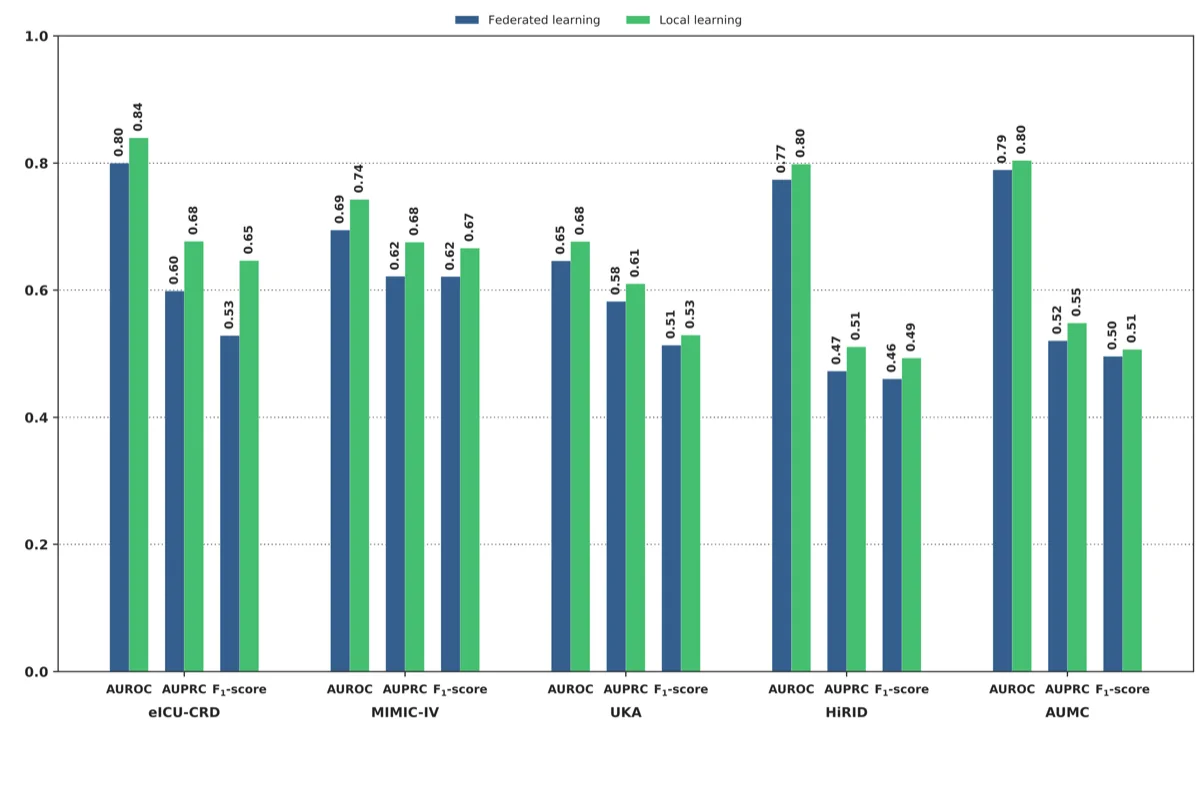
Federated vs local learning. Comparison of performance metrics across different datasets, showing the relative strengths of local learning. AUMC: Amsterdam University Medical Centers; AUPRC: area under the precision-recall curve; AUROC: area under the receiver operating characteristic curve; eICU-CRD: eICU Collaborative Research Database; HiRID: High-Resolution ICU Dataset; MIMIC-IV: Medical Information Mart for Intensive Care IV; UKA: Universitätsklinikum Augsburg.

Figure 3 compares the performance of the LL with the FL approaches; the LL model is trained and evaluated on each institution, whereas the FL model is trained in a federated approach on data from all institutions and evaluated on the data from each institution.

The performance comparison across institutions shows varying patterns. For eICU-CRD, the federated model achieved an AUROC of 0.80, AUPRC of 0.60, and *F*_1_-score of 0.53, while the LL model showed AUROC of 0.84, AUPRC of 0.68, and *F*_1_-score of 0.65. In MIMIC-IV, the federated model demonstrated AUROC of 0.69, AUPRC of 0.62, and *F*_1_-score of 0.62, compared to the LL model’s AUROC of 0.74, AUPRC of 0.68, and *F*_1_-score of 0.67. For UKA, the federated model achieved AUROC of 0.65, AUPRC of 0.58, and *F*_1_-score of 0.51, while the LL model showed AUROC of 0.68, AUPRC of 0.61, and *F*_1_-score of 0.53. In HiRID, the federated model demonstrated AUROC of 0.77, AUPRC of 0.47, and *F*_1_-score of 0.46, compared to the LL model’s AUROC of 0.80, AUPRC of 0.51, and *F*_1_-score of 0.49. For AUMC, the federated model achieved AUROC of 0.79, AUPRC of 0.52, and *F*_1_-score of 0.50; similarly, the LL model showed AUROC of 0.80, AUPRC of 0.55, and *F*_1_-score of 0.51. The LL models achieved higher performance metrics than the federated model across all databases, with AUROC gaps ranging from 0.01 (AUMC) to 0.05 (MIMIC-IV).

### FL vs CL Performance

**Figure 4:**
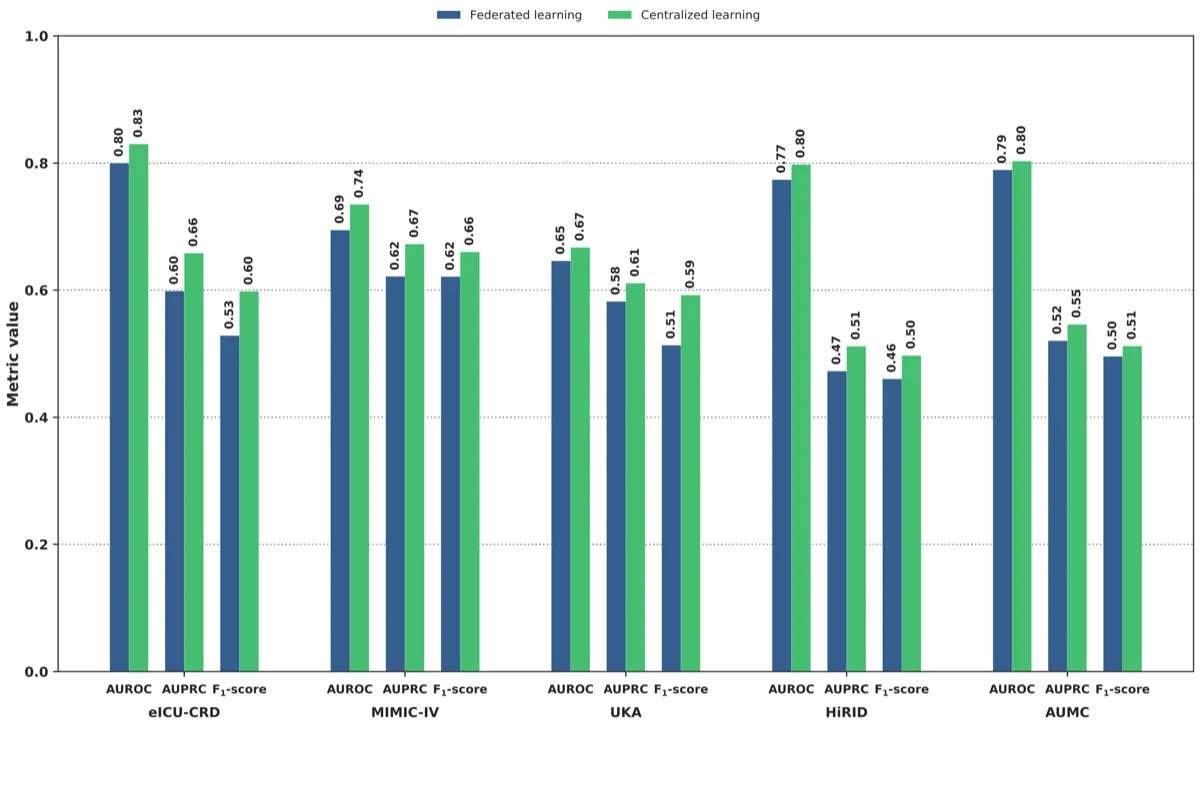
Federated vs centralized learning. Comparison of performance metrics across different datasets, showing relative strengths of centralized learning. AUMC: Amsterdam University Medical Centers; AUPRC: area under the precision-recall curve; AUROC: area under the receiver operating characteristic curve; eICU-CRD: eICU Collaborative Research Database; HiRID: High-Resolution ICU Dataset; MIMIC-IV: Medical Information Mart for Intensive Care IV; UKA: Universitätsklinikum Augsburg.

Figure 4 compares the performance of FL with CL models evaluated on each individual database. The CL model was trained on the pooled data from all institutions and then evaluated on each institution’s test dataset separately. For eICU-CRD, the federated model achieved an AUROC of 0.80, AUPRC of 0.60, and *F*_1_-score of 0.53, while the CL model showed AUROC of 0.83, AUPRC of 0.66, and *F*_1_-score of 0.60. In MIMIC-IV, the federated model achieved AUROC of 0.69, AUPRC of 0.62, and *F*_1_-score of 0.62, compared to the CL model’s AUROC of 0.74, AUPRC of 0.67, and *F*_1_-score of 0.66. For UKA, the federated model achieved AUROC of 0.65, AUPRC of 0.58, and *F*_1_-score of 0.51, while the CL model showed AUROC of 0.67, AUPRC of 0.61, and *F*_1_-score of 0.59. In HiRID, the federated model demonstrated AUROC of 0.77, AUPRC of 0.47, and *F*_1_-score of 0.46, compared to the CL model’s AUROC of 0.80, AUPRC of 0.51, and *F*_1_-score of 0.50. For AUMC, the federated model achieved AUROC of 0.79, AUPRC of 0.52, and *F*_1_-score of 0.50, while the CL model showed AUROC of 0.80, AUPRC of 0.55, and *F*_1_-score of 0.51.

Comparing site-level performance, the CL model achieved higher AUROC than the federated model at all 5 sites (eICU-CRD: 0.83 vs 0.80; MIMIC-IV: 0.74 vs 0.69; UKA: 0.67 vs 0.65; HiRID: 0.80 vs 0.77; AUMC: 0.80 vs 0.79), with gaps ranging from 0.01 (AUMC) to 0.05 (MIMIC-IV). These differences, while moderate, favored the CL approach and may reflect both the advantage of pooled training data and underlying dataset heterogeneity.

### Averaged FL vs Individual Institution

Figure S10 in Multimedia Appendix 1 compares the performance of the averaged FL model with individual institutions. The averaged FL model achieved an AUROC of 0.74, AUPRC of 0.56, and *F*_1_-score of 0.52. When compared to individual dataset models, the averaged federated model demonstrated lower AUROC values than 3 individual datasets: eICU-CRD (0.74 vs 0.80), HiRID (0.74 vs 0.77), and AUMC (0.74 vs 0.79). The averaged model outperformed MIMIC-IV and UKA individually, but fell below eICU-CRD, HiRID, and AUMC. For AUPRC, the individual dataset models from eICU-CRD (0.60), MIMIC-IV (0.62), and UKA (0.58) outperformed the averaged federated model (0.56), while HiRID (0.47) and AUMC (0.52) showed lower values. Similarly, for *F*_1_-scores, eICU-CRD (0.53) and MIMIC-IV (0.62) achieved higher values than the averaged federated model (0.52), while UKA (0.51), HiRID (0.46), and AUMC (0.50) showed comparable or lower values.

## Discussion

In this study, we built an FL approach to address this research gap, with the specific objectives of (1) evaluating the performance of FL compared to LL and CL approaches for MV weaning prediction, (2) assessing how model performance varies across diverse ICU databases, and (3) comparing predictive performance across 3 learning approaches that differ in their data-sharing requirements; privacy was not formally quantified and is therefore not framed as a measured trade-off.

### Principal Findings

Our study provides empirical evidence comparing FL, CL, and LL approaches for predicting successful weaning from MV across 5 diverse ICU databases. We observed the following key points:

First, we showed that, in this study, FL achieved reasonable predictive performance across 5 heterogeneous ICU databases without requiring the sharing of patient-level data across institutions. Federated models achieved AUROC values ranging from 0.65 (UKA) to 0.80 (eICU-CRD) across institutions, while CL models achieved AUROC values of 0.67 to 0.83. The federated model achieved this without directly accessing patient data, showing a measurable performance gap between our federated and CL models. As we did not formally measure privacy in this study, this gap should not be interpreted as quantifying a cost of privacy; rather, it reflects multiple factors inherent to this specific setup, including the bagging-based tree aggregation strategy, substantial differences in dataset sizes across sites, and cross-site heterogeneity in data distribution and documentation practices. A key factor influencing model performance was the substantial heterogeneity in our datasets. The proportion of positive outcomes varied dramatically across institutions: 35% (6169/17,466) in eICU-CRD, 46% (5196/11,367) in MIMIC-IV, 40% (3587/8973) in UKA, 18% (22,471/126,539) in HiRID, and 20% (11,579/58,932) in AUMC (based on overall episode-level outcome distributions). This variation represents a significant challenge for developing models across institutions.

Second, we observed substantial performance variation in LL models, with *F*_1_-scores ranging from 0.49 to 0.67 and AUPRC values from 0.51 to 0.68. These differences reflect several institutional factors rather than a single driver. Class distribution is one such factor: datasets with more balanced classes (eICU-CRD and MIMIC-IV) achieved higher AUPRC (0.68 and 0.68) and *F*_1_-scores (0.65 and 0.67) than those with imbalanced classes (HiRID: 18% positive, AUPRC=0.51; AUMC: 20% positive, AUPRC=0.55). However, because we applied a uniform 0.5 classification threshold across sites with widely different prevalences, part of the cross-site *F*_1_-score variation reflects threshold placement rather than model behavior itself; AUROC, which is threshold-independent, also varied across sites (0.68-0.84) and is affected by other factors. The dynamic weight adjustment described in the Methods partially mitigates the effect of class imbalance during local training, so we do not interpret class imbalance as the sole cause of cross-site differences. Alongside class distribution, case-mix heterogeneity, documentation frequency, temporal resolution, and feature-level missingness across sites all plausibly contribute, consistent with known challenges in FL on heterogeneous clinical data [22,35].

Third, LL models outperformed federated models at every site, with AUROC gaps ranging from 0.01 (AUMC) to 0.05 (MIMIC-IV); for example, eICU-CRD’s LL model achieved an *F*_1_-score of 0.65 vs 0.53 for the federated model. However, federated models achieved reasonable performance at every site (AUROC=0.65-0.80) without requiring patient-level data sharing, suggesting they may complement institution-specific models in scenarios such as limited local data, class imbalance, deployment on external cohorts, or settings where data-sharing constraints preclude pooled training. These scenarios were not directly tested in this study.

Finally, in this study, the CL model achieved a higher AUROC than the federated model at all 5 sites (gaps of 0.01 to 0.05). As only 1 CL and 1 federated configuration were evaluated, this observation should not be generalized to FL or CL more broadly.

### Cross-Silo FL in Health Care

Our study implements a cross-silo FL paradigm [36], in which multiple institutions collaborate on model development while minimizing direct data sharing. To support interoperability across institutions, we used the OMOP CDM to standardize variables across institutions, ensuring a shared feature space across all participating institutions. A key challenge in cross-silo FL is the coordination between institutions, particularly due to variability in data generation, preprocessing, and labeling practices. Our implementation addresses these challenges by enabling federated model training across 5 heterogeneous ICU databases, each characterized by distinct clinical practices, documentation standards, and outcome distributions. This shows the feasibility of cross-silo FL in real-world clinical settings, where data sharing is constrained by regulatory, ethical, and privacy requirements.

### Nonindependent and Nonidentically Distributed Data Challenges in Health Care

This study directly addresses the challenges posed by nonindependent and nonidentically distributed (non-IID) data in health care machine learning. The substantial variation in outcome distributions and clinical characteristics across institutions is known as a fundamental limitation in FL on clinical data [22,35]. In our study, this heterogeneity emerged primarily in 2 forms: feature distribution skew, where the input variables’ distribution differs across sites, and label distribution skew, characterized by varying proportions of positive outcomes. These differences are not merely statistical artifacts but originate from real variation in patient populations, clinical workflows, and documentation practices across institutions. Our implementation demonstrates that FL can accommodate such complexity by enabling collaborative model development without requiring data centralization. Despite these challenges, our federated approach achieved lower but reasonable AUROC values compared to those of CL models, indicating that it was able to capture some generalizable patterns across diverse clinical environments, though with a measurable performance gap. These findings offer empirical support for the feasibility of FL in real-world health care applications, even in the presence of significant data heterogeneity.

### Data Heterogeneity Impact on Model Performance

Beyond the class distribution differences noted above, our datasets exhibited additional dimensions of heterogeneity. Non-IID data in federated settings are commonly characterized along 3 axes: class distribution skew, feature distribution skew, and concept shift [36]. Class distribution skew is directly observable in the 18%-46% range of positive outcomes across sites, and feature distribution skew is apparent in the cross-site variation in variable distributions and correlation patterns (Tables S1-S5 and Figures S1-S6 in Multimedia Appendix 1). Differences in univariate feature-outcome associations across sites (Tables S1-S5 in Multimedia Appendix 1) are consistent with concept shift, though we did not formally test for it.

This heterogeneity in feature correlation represents what Kairouz et al [36] name as concept shift, where the relationship between features and outcomes differs across institutions. When models trained in federated settings encounter these varying patterns, they must learn to generalize across different feature relationships while still capturing institution-specific patterns that may be clinically relevant.

The performance differences observed across learning approaches in our study reflect the fundamental tension between local optimization and global generalization in federated systems. As shown in cross-institutional clinical FL studies [24], locally trained models can fully adapt to institution-specific patterns, often achieving strong performance within a single site; however, they risk overfitting to local variability and may generalize poorly. In this study, the federated model's site-level AUROC was below both the LL and CL values at every site, with gaps of 0.01 to 0.05. Whether a federated model balances performance across institutions is not a general property of FL; it depends on the aggregation strategy, underlying data distribution, and specific implementation choices. Different FL algorithms (eg, personalized FL and FedAvg variants) may produce qualitatively different behavior. CL models, meanwhile, optimize for overall aggregate performance but may overlook meaningful institution-specific differences. These trade-offs reflect the broader tension between privacy, use, and fairness in FL [36], emphasizing that the optimal approach depends on the clinical implementation context. Institutions with well-balanced, sufficiently large datasets may find locally trained models both effective and easier to deploy. In contrast, multi-institutional collaborations or environments where data-sharing constraints preclude pooled training may benefit more from FL, which can deliver reasonable predictive performance while avoiding direct sharing of patient data.

### Related Work

Despite advances in machine learning for ventilation weaning prediction, a significant research gap exists in developing models that can generalize across diverse clinical environments while respecting privacy regulations. Previous studies have primarily focused on single-institution data or CL approaches requiring data pooling, which limits their real-world applicability in health care systems where cross-institutional data sharing is restricted.

Our study extends previous research on machine learning applications in ventilator weaning prediction in several ways. Prior studies have demonstrated the effectiveness of various machine learning approaches in single-institution settings [13,14]. These studies primarily framed weaning as a binary classification task, typically predicting full weaning or extubation. Liao et al [13] used XGBoost to predict full weaning with an AUROC of 0.86, while Jia et al [14] achieved an AUROC of 0.94 using a convolutional neural network trained on MIMIC-III. Similarly, Liu et al [37] used data from MIMIC-IV and eICU-CRD to achieve AUROCs of 0.80 and 0.86, respectively. While existing studies by Otaguro et al [15] and Lin et al [16] show high performance (AUROCs of 0.95 and 0.91, respectively), they do not account for interinstitutional variability or data privacy constraints. In contrast to the prior studies, our study introduces an FL approach for MV weaning prediction across multiple institutions, thereby addressing the prevalent limitation of single-center generalizability.

Our study provides insights into the challenges and opportunities of FL under non-IID clinical data. The primary insights concern cross-site predictive performance and data heterogeneity; the privacy properties of federated tree-boosting approaches such as FedXgbBagging were not formally evaluated in this work. While the performance metrics achieved by our federated model (AUROC=0.74) are lower than those of several previous single-institution studies, they demonstrate that collaborative learning without data centralization can achieve reasonable results despite substantial data heterogeneity [18]. The direction of the performance gap we observed is broadly consistent with prior health care–FL studies and a systematic review [22,25,38], though the magnitude of such gaps varies with the FL algorithm, aggregation strategy, and dataset heterogeneity, so our gap should not be taken as representative of FL in general. Our results also provide evidence regarding the impact of dataset characteristics on model performance, particularly the relationship between class imbalance and prediction performance metrics.

### Limitations

This study evaluated a model based on as-is routine data from 5 institutions (eICU-CRD consists of data from multiple hospitals). Thus, by design, it includes various databases with a large heterogeneity in data collection practices and documentation standards across participating institutions, which may have introduced systematic biases that affect model performance. These variations in clinical practice and documentation have been identified as significant challenges in multi-institutional studies and FL implementations [13,36]. Our previous work has also highlighted how these institutional differences can impact model generalizability across different health care settings [18,19].

Substantial variations in temporal resolution and data completeness across institutions affected feature extraction quality. HiRID's high-frequency automated monitoring (2- to 5-minute intervals, 45%-55% episode completeness) contrasts with eICU-CRD's sparse multicenter documentation (208 hospitals, 10%-50% completeness), creating disparities in temporal pattern richness. Laboratory values showed particularly high variation in missingness (40%-100% depending on site and variable), reflecting different ordering practices and data capture systems. The 30% completeness threshold disproportionately excluded episodes from HiRID (826,392/952,931, 86.7%) compared to eICU-CRD (6309/23,775, 26.5%) and MIMIC-IV (1228/12,595, 9.7%); nonetheless, because HiRID contributed by far the largest pool of candidate episodes, it still dominated the final dataset. This may bias results toward high-frequency monitoring environments and raise concerns about equitable deployment across diverse health care settings. Additionally, feature importance patterns may reflect data availability rather than clinical relevance, as XGBoost tree-based splitting gives more opportunities to frequently measured variables than to intermittent ones, potentially creating artificial patterns based on documentation practices rather than true predictive value. Furthermore, our approach does not distinguish between clinically meaningful missingness (eg, tests not ordered due to clinical stability) and data capture missingness (eg, performed tests not recorded), as XGBoost treats all missing values uniformly, which may affect model interpretability and fairness across sites.

We did not exclude palliative care patients, whose PEEP reductions may reflect end-of-life care rather than successful weaning, potentially introducing misclassification bias. Furthermore, high missingness in PEEP documentation may have affected outcome label reliability, particularly in institutions where ventilator parameters were recorded primarily at setting changes. This limitation may have affected the accuracy of our positive outcome labels, particularly given that palliative care decisions and documentation practices vary significantly across institutions. The substantial variation in class distribution across institutions (18%-46% positive cases) is one of several sources of statistical heterogeneity that may limit generalizability and affect model convergence and fairness, alongside differences in case-mix, documentation frequency, temporal resolution, and feature-level missingness across sites [35].

We did not formally measure privacy or information leakage in this study. While the federated setup avoided sharing patient-level records, the serialized XGBoost tree structures exchanged under FedXgbBagging may still encode training-data information, which we did not evaluate under formal threat models (eg, membership inference) or mitigate with mechanisms such as differential privacy. Our findings should therefore be read as a comparison of predictive performance across approaches that differ in data-sharing requirements, not as a quantified privacy-performance trade-off. Future work should apply formal privacy accounting and evaluate defenses such as differential privacy or secure aggregation.

The federated setup also limited our ability to perform certain detailed error analyses and model interpretability studies that would require pooled data access, a limitation also noted in previous FL implementations in health care [23]. Our results highlight that LL models achieved the highest site-specific performance (AUROC 0.68-0.84). This may not generalize to settings with smaller local datasets or more imbalanced classes, where federated or CL models may perform more favorably. This suggests that the decision to implement FL vs LL approaches should consider not only data-sharing requirements but also the characteristics of local datasets. For deployment, additional quality control mechanisms such as site-specific performance monitoring and data quality-adjusted weighting schemes may be necessary to ensure trustworthy predictions across diverse clinical settings. Additionally, while we documented substantial heterogeneity in outcome prevalence and data quality across sites, detailed survival analyses (eg, Kaplan-Meier curves for mortality or time-to-event analyses) were beyond the scope of this prediction-focused study but could provide valuable insights in future work.

Demographic variables (age, gender, and BMI) were not included as model inputs, as the model was deliberately focused on time-varying physiological state. As age and gender are well-established predictors of weaning outcomes and differ substantially across sites (Table 2), their omission may have left unmeasured confounding in cross-site comparisons and could account for part of the observed performance heterogeneity that we otherwise attribute to documentation and case-mix differences. Future work should evaluate architectures that combine static demographics with time-series inputs.

### Conclusions

This study evaluated FL for MV weaning prediction across 5 ICU databases with 24,521 patients. LL models achieved the highest performance within their institutions (AUROC=0.68-0.84), while CL achieved an AUROC of 0.81 on pooled test data, and FL achieved a macroaveraged AUROC of 0.74 while avoiding direct data sharing.

Cross-site performance differences across all approaches likely reflected several interacting factors, including case-mix heterogeneity, documentation frequency, temporal resolution, feature-level missingness, and the interaction between class prevalence and the fixed 0.5 classification threshold; we did not isolate the contribution of each factor, and the dynamic weight adjustment applied during training partially mitigated the effect of class imbalance on local model fitting. The choice between LL, CL, and FL approaches in practice depends on local dataset size and balance, institutional data-sharing constraints, and acceptable performance thresholds for the intended clinical use. As we did not formally quantify privacy, our results should be interpreted as a performance comparison across approaches with different data-sharing requirements, rather than as a measured privacy-performance trade-off.

## Appendix

Multimedia Appendix 1 Supplementary materials including correlation analyses of clinical input variables across the 5 intensive care unit databases, data quality and missingness analyses, distributions of the 39 clinical input variables by weaning outcome for each database, per-site federated learning model performance, and confusion matrix counts by learning approach and site.

## Abbreviations

AUMC: Amsterdam University Medical Centers
AUPRC: area under the precision-recall curve
AUROC: area under the receiver operating characteristic curve
CDM: Common Data Model
CL: centralized learning
eICU-CRD: eICU Collaborative Research Database
FedAvg: federated averaging
FedXgbBagging: federated extreme gradient boosting bagging strategy
FL: federated learning
HiRID: High-Resolution ICU Dataset
ICU: intensive care unit
LL: local learning
LOINC: Logical Observation Identifiers Names and Codes
MIMIC-IV: Medical Information Mart for Intensive Care IV
MV: mechanical ventilation
non-IID: nonindependent and nonidentically distributed
OMOP: Observational Medical Outcomes Partnership
PEEP: positive end-expiratory pressure
SNOMED-CT: Systematized Nomenclature of Medicine-Clinical Terms
UKA: Universitätsklinikum Augsburg
XGBoost: extreme gradient boosting
