# Supplementary material for: Cross-Silo Federated Learning for Predicting Successful Mechanical Ventilation Weaning Across 5 Intensive Care Unit Databases: Retrospective Database Analysis
Source: JMIR Med Inform. 2026 Aug 14;14:e79713. doi: 10.2196/79713 (PMC13525363; doi:10.2196/79713)
Supplement: Supplementary file 1 [file medinform_v14i1e79713_app1.docx]

## Cross-Silo Federated Learning for Predicting Successful Mechanical Ventilation Weaning: A Study Across Five ICU Databases

Seyedmostafa Sheikhalishahi^1^*, Mathias Kaspar^1^*, Johanna Schwinn^1^, Matthaeus Morhart^1^, Philipp Simon^2^, Ludwig Christian Hinske^1^

^1^Digital Medicine, University Hospital of Augsburg, Augsburg, Germany

^2^Anesthesiology and Operative Intensive Care, Faculty of Medicine, University of Augsburg, Germany

*These authors contributed equally to this study.

**Corresponding Author:**

Seyedmostafa Sheikhalishahi

Email:seyedmostafa.sheikhalishahi@uk-augsburg.de

## Multimedia Appendix 1

### Introduction

This appendix provides supplementary materials for cross-silo federated learning study on predicting successful mechanical ventilation weaning across five intensive care unit (ICU) databases. Mechanical ventilation is a critical life-support intervention required by 50%-70% of ICU patients globally, but determining the optimal timing for weaning presents one of the most challenging decisions in critical care medicine.

Our main study evaluated the feasibility and efficacy of federated learning compared to local and centralized approaches for predicting successful reduction in Positive End-Expiratory Pressure (PEEP), a fundamental ventilation parameter reflecting respiratory recovery. The study utilized five diverse ICU databases: eICU Collaborative Research Database (eICU-CRD), Medical Information Mart for Intensive Care IV (MIMIC-IV), Universitätsklinikum Augsburg (UKA), High-Resolution ICU Dataset (HiRID), and Amsterdam University Medical Centers (AUMC).

We observed significant heterogeneity across these databases in terms of patient demographics, class distributions (18%-46% positive outcomes), and clinical practice patterns. The patient characteristics and data distribution variations create an ideal environment for evaluating different machine learning approaches.

This appendix provides detailed correlation analyses showing distinct patterns of relationships between clinical variables across the five databases, comprehensive patient characteristics stratified by weaning outcomes across all databases highlighting key differences in clinical parameters between positive and negative outcome groups, and comparative analysis of model performance between the averaged federated learning model and individual institution models. The detailed analyses presented here complement the main manuscript by illustrating the substantial heterogeneity across institutions and how our federated learning approach addresses this variability while avoiding direct sharing of patient data.

### Correlation Analysis

The five intensive care databases as shown in the Figure S1-S5 show generally consistent correlation patterns that reflect underlying physiological relationships, with some subtle differences between databases. All databases demonstrate expected strong correlations among related physiological parameters, such as the hematological indices MCH (Mean Corpuscular Hemoglobin), MCHC (Mean Corpuscular Hemoglobin Concentration), and MCV (Mean Corpuscular Volume), as well as between blood pressure measurements (MBP, DBP, SBP). eICU-CRD, MIMIC-IV, and HiRID show very similar correlation structures across most parameter pairs, suggesting robust physiological relationships that are consistent across different institutional settings and data collection protocols. The correlations between oxygen-related parameters (SaO_2_, PaO_2_, FiO_2_) and cardiovascular parameters maintain similar patterns across these three databases.

UKA exhibits generally weaker correlation coefficients across most parameter pairs compared to the other databases, which may be attributed to its smaller sample size (n=1,783 patients) or potentially greater patient heterogeneity within this cohort. Despite these weaker correlations, the overall pattern of relationships remains physiologically consistent.

A notable exception is observed in the AUMC database, where Albumin, Bilirubin, Chloride, and White Blood Cell count (WBC) show no correlations with any other variables (appearing as completely white columns in the correlation matrix). This absence of correlations reflects extremely high missing data rates for these parameters in AUMC.

Overall, while there are variations in correlation strength between databases and notable differences in data completeness (particularly in AUMC), the fundamental physiological relationships are preserved across datasets where sufficient data is available, supporting the validity of combining these databases for analytical purposes while accounting for institutional differences in data collection practices. Figure S6 presents the correlation matrix for the combined five ICU databases, showing the aggregate relationships across all institutions.

**Figure S1.** Correlation matrices of all clinical input variables for eICU-CRD; ALP: Alkaline Phosphatase; ALT: Alanine Aminotransferase; BMI: Body Mass Index; BUN: Blood Urea Nitrogen; DBP: Diastolic Blood Pressure; FiO2: Fraction of Inspired Oxygen; HR: Heart Rate; INR: International Normalized Ratio; MBP: Mean Blood Pressure; MCH: Mean Corpuscular Hemoglobin; MCHC: Mean Corpuscular Hemoglobin Concentration; MCV: Mean Corpuscular Volume; PEEP: Positive End-Expiratory Pressure; PIP: Peak Inspiratory Pressure; PaO_2_: Partial Pressure of Oxygen in Arterial Blood; RR: Respiratory Rate; SBP: Systolic Blood Pressure; SaO_2_: Oxygen Saturation in Arterial Blood; WBC: White Blood Cell Count; aPTT: Activated Partial Thromboplastin Time; pH: Potential of Hydrogen.

**Figure S2.** Correlation matrices of all clinical input variables for MIMIC-IV; ALP: Alkaline Phosphatase; ALT: Alanine Aminotransferase; BMI: Body Mass Index; BUN: Blood Urea Nitrogen; DBP: Diastolic Blood Pressure; FiO2: Fraction of Inspired Oxygen; HR: Heart Rate; INR: International Normalized Ratio; MBP: Mean Blood Pressure; MCH: Mean Corpuscular Hemoglobin; MCHC: Mean Corpuscular Hemoglobin Concentration; MCV: Mean Corpuscular Volume; PEEP: Positive End-Expiratory Pressure; PIP: Peak Inspiratory Pressure; PaO_2_: Partial Pressure of Oxygen in Arterial Blood; RR: Respiratory Rate; SBP: Systolic Blood Pressure; SaO_2_: Oxygen Saturation in Arterial Blood; WBC: White Blood Cell Count; aPTT: Activated Partial Thromboplastin Time; pH: Potential of Hydrogen.

**Figure S3.** Correlation matrices of all clinical input variables for UKA; ALP: Alkaline Phosphatase; ALT: Alanine Aminotransferase; BMI: Body Mass Index; BUN: Blood Urea Nitrogen; DBP: Diastolic Blood Pressure; FiO2: Fraction of Inspired Oxygen; HR: Heart Rate; INR: International Normalized Ratio; MBP: Mean Blood Pressure; MCH: Mean Corpuscular Hemoglobin; MCHC: Mean Corpuscular Hemoglobin Concentration; MCV: Mean Corpuscular Volume; PEEP: Positive End-Expiratory Pressure; PIP: Peak Inspiratory Pressure; PaO_2_: Partial Pressure of Oxygen in Arterial Blood; RR: Respiratory Rate; SBP: Systolic Blood Pressure; SaO_2_: Oxygen Saturation in Arterial Blood; WBC: White Blood Cell Count; aPTT: Activated Partial Thromboplastin Time; pH: Potential of Hydrogen.

**Figure S4.** Correlation matrices of all clinical input variables for HiRID; ALP: Alkaline Phosphatase; ALT: Alanine Aminotransferase; BMI: Body Mass Index; BUN: Blood Urea Nitrogen; DBP: Diastolic Blood Pressure; FiO2: Fraction of Inspired Oxygen; HR: Heart Rate; INR: International Normalized Ratio; MBP: Mean Blood Pressure; MCH: Mean Corpuscular Hemoglobin; MCHC: Mean Corpuscular Hemoglobin Concentration; MCV: Mean Corpuscular Volume; PEEP: Positive End-Expiratory Pressure; PIP: Peak Inspiratory Pressure; PaO_2_: Partial Pressure of Oxygen in Arterial Blood; RR: Respiratory Rate; SBP: Systolic Blood Pressure; SaO_2_: Oxygen Saturation in Arterial Blood; WBC: White Blood Cell Count; aPTT: Activated Partial Thromboplastin Time; pH: Potential of Hydrogen.

**Figure S5.** Correlation matrices of all clinical input variables for AUMC; ALP: Alkaline Phosphatase; ALT: Alanine Aminotransferase; BMI: Body Mass Index; BUN: Blood Urea Nitrogen; DBP: Diastolic Blood Pressure; FiO2: Fraction of Inspired Oxygen; HR: Heart Rate; INR: International Normalized Ratio; MBP: Mean Blood Pressure; MCH: Mean Corpuscular Hemoglobin; MCHC: Mean Corpuscular Hemoglobin Concentration; MCV: Mean Corpuscular Volume; PEEP: Positive End-Expiratory Pressure; PIP: Peak Inspiratory Pressure; PaO_2_: Partial Pressure of Oxygen in Arterial Blood; RR: Respiratory Rate; SBP: Systolic Blood Pressure; SaO_2_: Oxygen Saturation in Arterial Blood; WBC: White Blood Cell Count; aPTT: Activated Partial Thromboplastin Time; pH: Potential of Hydrogen.

**Figure S6.** Correlation matrices of all clinical input variables for combined five icu databases; ALP: Alkaline Phosphatase; ALT: Alanine Aminotransferase; BMI: Body Mass Index; BUN: Blood Urea Nitrogen; DBP: Diastolic Blood Pressure; FiO2: Fraction of Inspired Oxygen; HR: Heart Rate; INR: International Normalized Ratio; MBP: Mean Blood Pressure; MCH: Mean Corpuscular Hemoglobin; MCHC: Mean Corpuscular Hemoglobin Concentration; MCV: Mean Corpuscular Volume; PEEP: Positive End-Expiratory Pressure; PIP: Peak Inspiratory Pressure; PaO_2_: Partial Pressure of Oxygen in Arterial Blood; RR: Respiratory Rate; SBP: Systolic Blood Pressure; SaO_2_: Oxygen Saturation in Arterial Blood; WBC: White Blood Cell Count; aPTT: Activated Partial Thromboplastin Time; pH: Potential of Hydrogen.

### Data Quality and Missingness Analysis

This section provides comprehensive documentation of data quality variations across the five participating ICU databases. Understanding these patterns is essential for interpreting federated learning performance and assessing the reliability of model predictions across heterogeneous institutional settings. Figures S7-S9 document data quality variations across the five ICU databases. Figure S7 shows episode-level completeness distributions with the 30% threshold (red dashed line): HiRID demonstrates concentrated completeness (45-55%) from automated monitoring, eICU-CRD shows bimodal patterns (10-50%) reflecting multi-center heterogeneity, and MIMIC-IV displays broad distribution (10-40%). Figure S8 presents feature-specific missingness heatmaps revealing systematic institutional differences: laboratory values showed highest variation (albumin: 42-99% missing; bilirubin: 15-97% missing), vital signs varied substantially (heart rate: 24-92% missing), and ventilator parameters showed paradoxically high missingness (PEEP, PIP, FiO2) likely reflecting documentation primarily at setting changes. Figure S9 illustrates temporal missingness patterns across 24-hour observation windows, showing consistent increases in missingness as events approach (50-65% at 24 hours before to 70-92% at event time) across all sites, reflecting clinical workflow shifts toward intervention rather than routine documentation. HiRID maintained lowest missingness (50-58%) throughout due to high-frequency automated monitoring, while eICU-CRD and MIMIC-IV showed steeper increases. Given these patterns, we used XGBoost’s native missing value handling rather than imputation.

**Figure S7.** Distribution of episode completeness across five ICU databases. The red dashed line indicates the 30% completeness threshold below which episodes were excluded from analysis.

**Figure S8.** Heatmap of feature-specific missingness patterns across five ICU databases. ALP: Alkaline Phosphatase; ALT: Alanine Aminotransferase; aPTT: Activated Partial Thromboplastin Time; AUMC: Amsterdam University Medical Centers; BMI: Body Mass Index; BUN: Blood Urea Nitrogen; DBP: Diastolic Blood Pressure; eICU-CRD: eICU Collaborative Research Database; FiO_2_: Fraction of Inspired Oxygen; HiRID: High-Resolution ICU Dataset; HR: Heart Rate; ICU: intensive care unit; INR: International Normalized Ratio; MBP: Mean Blood Pressure; MCH: Mean Corpuscular Hemoglobin; MCHC: Mean Corpuscular Hemoglobin Concentration; MCV: Mean Corpuscular Volume; MIMIC-IV: Medical Information Mart for Intensive Care; PaO_2_: Partial Pressure of Oxygen in Arterial Blood; PEEP: Positive End-Expiratory Pressure; pH: Potential of Hydrogen; PIP: Peak Inspiratory Pressure; RR: Respiratory Rate; SaO2: Oxygen Saturation in Arterial Blood; SBP: Systolic Blood Pressure; UKA: Universitätsklinikum Augsburg; WBC: White Blood Cell Count.

**Figure S9.** Temporal evolution of data missingness in 24-hour observation windows preceding PEEP reduction events.

**Distribution of Clinical Input Variables by Outcome**

Tables S1-S5 describe the distributions of the 39 clinical variables used as model inputs, stratified by weaning outcome.

#### eICU-CRD - Input Variable Distribution

As shown in Table S1 eICU-CRD database, patients with positive outcomes showed significantly higher hemoglobin (10.1 vs. 9.6 g/dL, *P*<.001), albumin (2.6 vs. 2.3 g/dL, *P*<.001), and oxygen levels (PaO_2_: 130.4 vs. 117.2 mmHg, *P*<.001). They also showed lower alkaline phosphatase, aPTT, INR, and urea nitrogen levels compared to the negative outcome group.

**Table S1**. Distribution of the 39 clinical input variables by weaning outcome in eICU-CRD.^a^

| **Clinical Variable** | | **Positive outcome** | **Negative outcome** | ***P*-value** |
| --- | --- | --- | --- | --- |
| Sodium (Moles/volume) | | 141.1 (4.4) | 140.9 (5.0) | .279 |
| Hemoglobin (Mass/volume) | | 10.1 (1.8) | 9.6 (1.8) | < .001 |
| Alkaline phosphatase (Enzymatic activity/volume) | | 100.3 (57.5) | 121.6 (80.7) | .004 |
| MCHC (Mass/volume) | | 33.1 (1.3) | 33.0 (1.3) | .217 |
| Potassium (Moles/volume) | | 4.0 (0.5) | 3.9 (0.5) | < .001 |
| Bilirubin.total (Moles/volume) | | 1.3 (1.5) | 1.7 (1.5) | .057 |
| Bicarbonate (Moles/volume) | | 24.7 (4.3) | 25.0 (4.4) | .064 |
| Platelets (#/volume) | | 179.1 (87.3) | 173.3 (99.4) | .019 |
| Base excess in Blood by calculation | | 3.9 (3.4) | 4.7 (3.9) | < .001 |
| aPTT in Blood by Coagulation assay | | 39.9 (17.3) | 45.2 (20.2) | < .001 |
| Chloride (Moles/volume) | | 106.9 (5.6) | 106.4 (6.0) | .025 |
| pH of Arterial blood | | 7.4 (0.1) | 7.4 (0.1) | .731 |
| Glucose (Moles/volume) | | 143.7 (43.1) | 147.5 (45.1) | .001 |
| Body temperature | | 37.2 (0.6) | 37.3 (0.6) | < .001 |
| MCV (Entitic volume) | | 90.2 (5.1) | 90.3 (5.5) | .880 |
| Albumin (Mass/volume) | | 2.6 (0.6) | 2.3 (0.5) | < .001 |
| Oxygen (Partial pressure) | | 130.4 (69.3) | 117.2 (64.8) | < .001 |
| Calcium | | 8.1 (0.6) | 8.1 (0.7) | .758 |
| INR in Blood by Coagulation assay | | 1.4 (0.5) | 1.5 (0.5) | .036 |
| MCH (Entitic mass) | | 29.8 (2.0) | 29.7 (1.9) | .526 |
| Anion gap | | 10.9 (3.8) | 11.5 (3.6) | .001 |
| Aspartate aminotransferase | | 83.8 (121.5) | 99.7 (153.9) | .621 |
| Peak inspiratory pressure | | 24.4 (6.2) | 24.5 (6.4) | .819 |
| Systolic blood pressure | | 120.3 (19.7) | 119.8 (19.4) | .140 |
| Mean blood pressure | | 79.4 (13.1) | 78.7 (12.9) | .014 |
| Heart rate | | 86.2 (16.4) | 87.8 (16.6) | < .001 |
| White blood cell count | | 12.8 (5.0) | 12.7 (5.7) | .258 |
| Respiratory rate | | 19.5 (5.3) | 19.5 (5.3) | .889 |
| Positive end expiratory pressure | | 9.1 (2.6) | 6.9 (2.4) | < .001 |
| Inspired oxygen concentration | | 57.3 (22.7) | 57.0 (22.1) | .427 |
| Invasive diastolic arterial pressure | | 59.7 (11.1) | 59.8 (11.0) | .867 |
| Arterial oxygen saturation | | 96.8 (3.3) | 95.3 (4.5) | < .001 |
| Lactate (Mass/volume) | | 2.6 (2.3) | 3.0 (2.6) | .005 |
| Urea nitrogen (Moles/volume) | | 10.1 (6.3) | 11.7 (7.2) | .001 |
| Creatinine (Moles/volume) | | 1.5 (1.0) | 1.5 (1.1) | .671 |
| Alanine aminotransferase (Enzymatic activity/volume) | | 79.8 (109.9) | 86.5 (130.2) | .910 |
| Glasgow Coma Score | Motor (Median) | 6.0 | 6.0 | .007 |
|  | Verbal (Median) | 1.0 | 1.0 | .280 |
|  | Eye (Median) | 4.0 | 4.0 | .749 |

^a^ All values are represented in mean (SD), otherwise specified. *P*-values were calculated by χ2-test or Wilcoxon-Mann-Whitney test, as appropriate. aPTT: Activated Partial Thromboplastin Time; INR: International Normalized Ratio; MCH: Mean Corpuscular Hemoglobin; MCHC: Mean Corpuscular Hemoglobin Concentration; MCV: Mean Corpuscular Volume; SD: Standard Deviation.

#### MIMIC-IV - Input Variable Distribution

As shown in Table S2, similar to eICU-CRD, MIMIC-IV patients with positive weaning outcomes demonstrated better oxygenation (PaO_2_: 124.8 vs. 111.5 mmHg, *P*<.001), higher hemoglobin (9.7 vs. 9.4 g/dL, *P*=.001), and higher baseline PEEP. Notable difference from eICU-CRD include stronger associations with sodium levels (139.0 vs. 139.8 mmol/L, P=.001).

**Table S2**. Distribution of the 39 clinical input variables by weaning outcome in MIMIC-IV.^b^

| **Clinical Variable** | | **Positive outcome** | **Negative outcome** | ***P*-value** |
| --- | --- | --- | --- | --- |
| Sodium (Moles/volume) | | 139.0 (4.4) | 139.8 (4.7) | .001 |
| Hemoglobin (Mass/volume) | | 9.7 (1.7) | 9.4 (1.6) | .001 |
| Alkaline phosphatase (Enzymatic activity/volume) | | 121.5 (83.5) | 126.9 (84.2) | .404 |
| MCHC (Mass/volume) | | 33.1 (1.5) | 32.6 (1.5) | < .001 |
| Potassium (Moles/volume) | | 4.1 (0.5) | 4.1 (0.5) | .435 |
| Bilirubin.total (Moles/volume) | | 2.8 (3.9) | 2.9 (4.1) | .890 |
| Bicarbonate (Moles/volume) | | 24.2 (3.9) | 24.6 (4.5) | .208 |
| Platelets (#/volume) | | 183.3 (109.1) | 189.8 (117.1) | .531 |
| Base excess in Blood by calculation | | 0.2 (4.0) | 0.4 (4.4) | .115 |
| aPTT in Blood by Coagulation assay | | 41.9 (19.3) | 40.9 (17.2) | .769 |
| Chloride (Moles/volume) | | 103.5 (5.2) | 103.7 (5.7) | .337 |
| pH of Arterial blood | | 7.4 (0.1) | 7.4 (0.1) | .558 |
| Glucose (Moles/volume) | | 138.1 (39.4) | 141.8 (44.2) | .087 |
| Body temperature | | 37.2 (0.6) | 37.2 (0.6) | .174 |
| MCV (Entitic volume) | | 91.4 (5.9) | 91.8 (5.9) | .188 |
| Albumin (Mass/volume) | | 2.9 (0.5) | 2.8 (0.5) | .165 |
| Oxygen (Partial pressure) | | 124.8 (46.4) | 111.5 (41.0) | < .001 |
| Calcium | | 4.6 (3.6) | 6.1 (3.4) | < .001 |
| INR in Blood by Coagulation assay | | 1.4 (0.4) | 1.5 (0.5) | .913 |
| MCH (Entitic mass) | | 30.2 (1.9) | 29.9 (2.1) | .173 |
| Anion gap | | 14.5 (3.4) | 14.1 (3.6) | .034 |
| Aspartate aminotransferase | | 108.4 (126.6) | 88.7 (108.5) | .352 |
| Peak inspiratory pressure | | 21.0 (6.1) | 18.2 (6.5) | < .001 |
| Systolic blood pressure | | 117.1 (18.0) | 118.4 (18.9) | .001 |
| Mean blood pressure | | 77.2 (12.1) | 77.2 (12.7) | .335 |
| Heart rate | | 84.1 (15.2) | 85.1 (15.8) | .001 |
| White blood cell count | | 12.7 (5.2) | 12.2 (5.1) | .040 |
| Respiratory rate | | 14.6 (9.3) | 16.1 (8.9) | < .001 |
| Positive end expiratory pressure | | 9.9 (2.8) | 7.7 (2.8) | < .001 |
| Inspired oxygen concentration | | 49.8 (14.5) | 47.5 (13.4) | .021 |
| Invasive diastolic arterial pressure | | 59.1 (10.4) | 58.2 (11.2) | < .001 |
| Arterial oxygen saturation | | 93.2 (10.3) | 92.8 (10.6) | .082 |
| Lactate (Mass/volume) | | 1.9 (1.2) | 1.9 (1.3) | .123 |
| Urea nitrogen (Moles/volume) | | 10.9 (6.6) | 11.6 (6.9) | .028 |
| Creatinine (Moles/volume) | | 1.6 (1.1) | 1.6 (1.2) | .372 |
| Alanine aminotransferase (Enzymatic activity/volume) | | 89.9 (130.3) | 74.5 (100.0) | .487 |
| Glasgow Coma Score | Motor (Median) | 6.0 | 6.0 | < .001 |
|  | Verbal (Median) | 1.0 | 1.0 | .600 |
|  | Eye (Median) | 3.0 | 3.0 | .163 |

^b^ All values are represented in mean (SD), otherwise specified. P-values were calculated by χ2-test or Wilcoxon-Mann-Whitney test, as appropriate. aPTT: Activated Partial Thromboplastin Time; INR: International Normalized Ratio; MCH: Mean Corpuscular Hemoglobin; MCHC: Mean Corpuscular Hemoglobin Concentration; MCV: Mean Corpuscular Volume; SD: Standard Deviation.

#### UKA - Input Variable Distribution

As shown in Table S3, UKA database showed unique patterns compared to other institutions, with less pronounced differences in laboratory values between outcome groups. Significant differences were observed in base excess (-0.6 vs. 0.1 mmol/L, *P*<.001), and oxygen partial pressure (91.9 vs. 86.1 mmHg, *P*<.001). Unlike other databases, parameters such as albumin, bilirubin, calcium, and several other laboratory values showed no significant difference between outcome groups.

**Table S3**. Distribution of the 39 clinical input variables by weaning outcome in UKA.^c^

| **Clinical Variable** | | **Positive outcome** | **Negative outcome** | ***P*-value** |
| --- | --- | --- | --- | --- |
| Sodium (Moles/volume) | | 141.3 (4.7) | 141.7 (4.9) | .013 |
| Hemoglobin (Mass/volume) | | 10.3 (2.1) | 10.1 (2.2) | .018 |
| Alkaline phosphatase (Enzymatic activity/volume) | | 127.5 (89.0) | 111.9 (56.4) | .886 |
| MCHC (Mass/volume) | | 33.4 (1.4) | 33.0 (1.3) | .006 |
| Potassium (Moles/volume) | | 4.2 (0.5) | 4.2 (0.5) | .652 |
| Bilirubin.total (Moles/volume) | | 1.2 (1.4) | 1.0 (1.2) | .601 |
| Bicarbonate (Moles/volume) | | 22.4 (3.1) | 23.0 (3.5) | .458 |
| Platelets (#/volume) | | 204.3 (115.2) | 216.9 (117.4) | .278 |
| Base excess in Blood by calculation | | -0.6 (4.3) | 0.1 (4.4) | < .001 |
| aPTT in Blood by Coagulation assay | | 41.6 (13.9) | 41.8 (12.0) | .266 |
| Chloride (Moles/volume) | | 110.0 (5.7) | 109.8 (6.1) | .241 |
| pH of Arterial blood | | 7.4 (0.1) | 7.4 (0.1) | .409 |
| Glucose (Moles/volume) | | 159.5 (45.8) | 162.0 (46.0) | .124 |
| Body temperature | | 37.3 (0.7) | 37.3 (0.7) | .033 |
| MCV (Entitic volume) | | 90.7 (5.5) | 91.3 (5.5) | .347 |
| Albumin (Mass/volume) | | 2.9 (0.4) | 2.8 (0.5) | .077 |
| Oxygen (Partial pressure) | | 91.9 (31.1) | 86.1 (26.7) | < .001 |
| Calcium | | 2.2 (0.2) | 2.2 (0.2) | .219 |
| INR in Blood by Coagulation assay | | 1.2 (0.3) | 1.1 (0.2) | .030 |
| MCH (Entitic mass) | | 30.3 (1.9) | 30.1 (1.9) | .295 |
| Anion gap | | 6.6 (3.8) | 6.6 (3.6) | .409 |
| Aspartate aminotransferase | | 90.3 (157.9) | 80.4 (87.9) | .245 |
| Peak inspiratory pressure | | 13.5 (4.2) | 14.1 (4.4) | < .001 |
| Systolic blood pressure | | 120.7 (20.5) | 120.4 (20.0) | .889 |
| Mean blood pressure | | 83.6 (14.6) | 82.1 (14.7) | .361 |
| Heart rate | | 83.9 (16.5) | 83.9 (16.9) | .970 |
| White blood cell count | | 12.3 (5.6) | 12.6 (5.5) | .480 |
| Respiratory rate | | 17.2 (4.7) | 18.8 (5.2) | < .001 |
| Positive end expiratory pressure | | 10.9 (2.7) | 10.6 (2.7) | < .001 |
| Inspired oxygen concentration | | 41.7 (15.6) | 44.6 (17.0) | < .001 |
| Invasive diastolic arterial pressure | | 59.8 (10.8) | 59.3 (10.6) | .042 |
| Arterial oxygen saturation | | 93.5 (8.7) | 93.3 (7.8) | < .001 |
| Lactate (Mass/volume) | | 1.5 (1.0) | 1.5 (0.9) | .952 |
| Urea nitrogen (Moles/volume) | | 25.5 (14.1) | 27.8 (13.9) | .066 |
| Creatinine (Moles/volume) | | 1.4 (0.9) | 1.5 (0.9) | .601 |
| Alanine aminotransferase (Enzymatic activity/volume) | | 67.7 (79.9) | 64.7 (82.1) | .695 |
| Glasgow Coma Score | Motor (Median) | 1.0 | 1.0 | .220 |
|  | Verbal (Median) | 1.0 | 1.0 | .132 |
|  | Eye (Median) | 4.0 | 1.0 | .426 |

^c^ All values are represented in mean (SD), otherwise specified. P-values were calculated by χ2-test or Wilcoxon-Mann-Whitney test, as appropriate. aPTT: Activated Partial Thromboplastin Time; INR: International Normalized Ratio; MCH: Mean Corpuscular Hemoglobin; MCHC: Mean Corpuscular Hemoglobin Concentration; MCV: Mean Corpuscular Volume; SD: Standard Deviation.

#### HiRID - Input Variable Distribution

As shown in Table S4, HiRID shows associations between positive outcomes with significantly higher hemoglobin (9.9 vs. 9.7 g/dL, *P*<.001), potassium (4.2 vs. 4.1 mmol/L, *P* <.001), and oxygenation values (PaO_2_: 96.3 vs. 91.0 mmHg, *P*<.001).

**Table S4**. Distribution of the 39 clinical input variables by weaning outcome in HiRID.^d^

| **Clinical Variable** | | **Positive outcome** | **Negative outcome** | ***P*-value** |
| --- | --- | --- | --- | --- |
| Sodium (Moles/volume) | | 138.3 (4.6) | 138.3 (4.9) | .998 |
| Hemoglobin (Mass/volume) | | 9.9 (1.5) | 9.7 (1.4) | < .001 |
| Alkaline phosphatase (Enzymatic activity/volume) | | 131.9 (97.2) | 151.8 (103.6) | < .001 |
| MCHC (Mass/volume) | | 33.6 (0.9) | 33.7 (1.0) | < .001 |
| Potassium (Moles/volume) | | 4.2 (0.5) | 4.1 (0.5) | < .001 |
| Bilirubin.total (Moles/volume) | | 1.6 (2.5) | 2.1 (3.1) | < .001 |
| Bicarbonate (Moles/volume) | | 24.8 (3.3) | 25.2 (3.4) | < .001 |
| Platelets (#/volume) | | 192.6 (122.3) | 182.9 (125.0) | < .001 |
| Base excess in Blood by calculation | | 0.8 (3.6) | 1.2 (3.6) | < .001 |
| aPTT in Blood by Coagulation assay | | 43.0 (13.2) | 46.5 (13.7) | < .001 |
| Chloride (Moles/volume) | | 107.9 (4.4) | 107.8 (4.8) | < .001 |
| pH of Arterial blood | | 7.4 (0.1) | 7.4 (0.1) | < .001 |
| Glucose (Moles/volume) | | 154.1 (36.6) | 153.8 (35.3) | .619 |
| Body temperature | | 37.3 (0.7) | 37.2 (0.8) | < .001 |
| MCV (Entitic volume) | | 91.1 (4.2) | 91.2 (4.5) | .201 |
| Albumin (Mass/volume) | | 1.9 (0.4) | 1.8 (0.5) | .003 |
| Oxygen (Partial pressure) | | 96.3 (29.7) | 91.0 (26.5) | < .001 |
| Calcium | | 1.2 (0.1) | 1.1 (0.1) | < .001 |
| INR in Blood by Coagulation assay | | 1.2 (0.2) | 1.2 (0.2) | .419 |
| MCH (Entitic mass) | | 30.6 (1.5) | 30.8 (1.6) | < .001 |
| Anion gap | | 5.9 (3.9) | 5.8 (3.8) | .076 |
| Aspartate aminotransferase | | 166.4 (351.0) | 184.3 (379.8) | .006 |
| Peak inspiratory pressure | | 24.4 (6.8) | 23.5 (6.6) | < .001 |
| Systolic blood pressure | | 114.0 (23.8) | 115.7 (23.8) | < .001 |
| Mean blood pressure | | 74.1 (14.2) | 74.5 (14.2) | < .001 |
| Heart rate | | 87.7 (14.8) | 89.2 (15.5) | < .001 |
| White blood cell count | | 12.1 (5.0) | 12.2 (5.5) | .344 |
| Respiratory rate | | 20.2 (10.9) | 20.6 (7.9) | < .001 |
| Positive end expiratory pressure | | 7.6 (2.2) | 6.8 (2.1) | < .001 |
| Inspired oxygen concentration | | 45.1 (14.9) | 44.0 (13.4) | < .001 |
| Invasive diastolic arterial pressure | | 55.0 (10.5) | 55.0 (10.6) | .445 |
| Arterial oxygen saturation | | 96.9 (2.3) | 96.4 (2.5) | < .001 |
| Lactate (Mass/volume) | | 1.7 (1.1) | 1.8 (1.1) | < .001 |
| Urea nitrogen (Moles/volume) | | 12.3 (7.2) | 12.0 (6.8) | .342 |
| Creatinine (Moles/volume) | | 1.3 (0.9) | 1.4 (0.9) | .363 |
| Alanine aminotransferase (Enzymatic activity/volume) | | 131.8 (217.5) | 129.0 (235.6) | .163 |
| Glasgow Coma Score | Motor (Median) | 6.0 | 6.0 | < .001 |
|  | Verbal (Median) | 1.0 | 4.0 | < .001 |
|  | Eye (Median) | 4.0 | 4.0 | < .001 |

^d^ All values are represented in mean (SD), otherwise specified. P-values were calculated by χ2-test or Wilcoxon-Mann-Whitney test, as appropriate. aPTT: Activated Partial Thromboplastin Time; INR: International Normalized Ratio; MCH: Mean Corpuscular Hemoglobin; MCHC: Mean Corpuscular Hemoglobin Concentration; MCV: Mean Corpuscular Volume; SD: Standard Deviation.

#### AUMC - Input Variable Distribution

As shown in Table S5, despite several variables being unavailable in this database AUMC database showed consistent patterns with other institutions regarding the association between positive outcomes and better oxygenation (PaO_2_: 111.6 vs. 98.9 mmHg, *P*<0.001), higher hemoglobin levels (10.5 vs. 10.0 g/dL, *P*<.001), and lower urea nitrogen (10.8 vs. 13.3 mmol/L, *P*<.001). Unique to AUMC was a stronger association between positive outcomes and lower aPTT values (46.5 vs. 54.1 seconds, *P*<.001) and significantly higher glucose levels in the positive outcome group (143.8 vs. 133.5 mg/dL, *P*<.001).

**Table S5**. Distribution of the 39 clinical input variables by weaning outcome in AUMC.^e^

| **Clinical Variable** | | **Positive outcome** | **Negative outcome** | ***P*-value** |
| --- | --- | --- | --- | --- |
| Sodium (Moles/volume) | | 141.8 (4.3) | 142.6 (5.1) | .001 |
| Hemoglobin (Mass/volume) | | 10.5 (1.7) | 10.0 (1.6) | < .001 |
| Alkaline phosphatase (Enzymatic activity/volume) | | 114.0 (87.4) | 137.7 (101.6) | < .001 |
| MCHC (Mass/volume) | | 21.0 (0.8) | 21.2 (0.9) | .313 |
| Potassium (Moles/volume) | | 4.2 (0.4) | 4.2 (0.4) | .040 |
| Bilirubin.total (Moles/volume) | | N/A | N/A | N/A |
| Bicarbonate (Moles/volume) | | 24.4 (4.1) | 25.6 (4.4) | < .001 |
| Platelets (#/volume) | | 187.1 (104.9) | 199.9 (127.9) | .200 |
| Base excess in Blood by calculation | | 2.5 (3.6) | 3.0 (3.7) | < .001 |
| aPTT in Blood by Coagulation assay | | 46.5 (16.1) | 54.1 (18.3) | < .001 |
| Chloride (Moles/volume) | | N/A | N/A | N/A |
| pH of Arterial blood | | 7.4 (0.1) | 7.4 (0.1) | < .001 |
| Glucose (Moles/volume) | | 143.8 (36.1) | 133.5 (32.7) | < .001 |
| Body temperature | | 36.3 (1.3) | 36.4 (1.4) | < .001 |
| MCV (Entitic volume) | | 91.9 (4.8) | 90.2 (4.8) | .033 |
| Albumin (Mass/volume) | | N/A | N/A | N/A |
| Oxygen (Partial pressure) | | 111.6 (35.5) | 98.9 (29.5) | < .001 |
| Calcium | | 2.0 (0.2) | 2.0 (0.2) | .062 |
| INR in Blood by Coagulation assay | | 1.4 (0.3) | 1.4 (0.3) | < .001 |
| MCH (Entitic mass) | | 31.0 (1.9) | 30.6 (1.7) | .217 |
| Anion gap | | 8.7 (3.1) | 8.6 (3.2) | .065 |
| Aspartate aminotransferase | | 136.1 (215.1) | 138.6 (203.0) | .151 |
| Peak inspiratory pressure | | 22.1 (7.0) | 22.3 (7.2) | .001 |
| Systolic blood pressure | | 127.6 (23.7) | 130.4 (23.6) | < .001 |
| Mean blood pressure | | 83.0 (14.1) | 82.7 (13.8) | .123 |
| Heart rate | | 85.5 (17.1) | 88.4 (17.5) | < .001 |
| White blood cell count | | N/A | N/A | N/A |
| Respiratory rate | | 18.8 (5.2) | 20.4 (5.4) | < .001 |
| Positive end expiratory pressure | | 10.8 (3.2) | 10.3 (3.1) | < .001 |
| Inspired oxygen concentration | | 44.2 (8.1) | 45.1 (8.4) | < .001 |
| Invasive diastolic arterial pressure | | 61.7 (11.2) | 60.6 (11.0) | < .001 |
| Arterial oxygen saturation | | 85.5 (31.9) | 87.1 (29.6) | < .001 |
| Lactate (Mass/volume) | | 2.0 (1.5) | 2.0 (1.5) | .693 |
| Urea nitrogen (Moles/volume) | | 10.8 (6.4) | 13.3 (7.4) | < .001 |
| Creatinine (Moles/volume) | | 1.3 (0.7) | 1.4 (0.8) | < .001 |
| Alanine aminotransferase (Enzymatic activity/volume) | | 100.4 (145.5) | 113.1 (160.2) | .003 |
| Glasgow Coma Score | Motor (Median) | 6.0 | 6.0 | < .001 |
|  | Verbal (Median) | 1.0 | 1.0 | .032 |
|  | Eye (Median) | 4.0 | 1.0 | < .001 |

^e^ All values are represented in mean (SD), otherwise specified. P-values were calculated by χ2-test or Wilcoxon-Mann-Whitney test, as appropriate. aPTT: Activated Partial Thromboplastin Time; INR: International Normalized Ratio; MCH: Mean Corpuscular Hemoglobin; MCHC: Mean Corpuscular Hemoglobin Concentration; MCV: Mean Corpuscular Volume; SD: Standard Deviation.

### Model Performance Analysis

Figure S10 compares performance metrics (AUROC, AUPRC, F1-score) between the averaged federated learning model and the federated model evaluated on each site’s test data. The averaged federated model (0.74 AUROC) outperforms MIMIC-IV (0.69) and UKA (0.65), but underperforms compared to eICU-CRD (0.80), HiRID (0.77), and AUMC (0.79). For AUPRC, MIMIC-IV (0.62), eICU-CRD (0.60), and UKA (0.58) achieve higher values than the averaged federated model (0.56), while HiRID (0.47) and AUMC (0.52) show lower values. For F1-scores, MIMIC-IV (0.62) and eICU-CRD (0.53) exceed the averaged federated model (0.52), while UKA (0.51), AUMC (0.50), and HiRID (0.46) show comparable or lower values. Cross-site differences in AUPRC and F1 reflect multiple factors, including class distribution at our fixed 0.5 classification threshold, alongside case-mix heterogeneity, documentation frequency, and feature missingness.

**Figure S10.** Comparison of averaged federated learning performance with per-site federated model performance. eICU-CRD: eICU Collaborative Research Database; MIMIC-IV: Medical Information Mart for Intensive Care; UKA: Universitätsklinikum Augsburg; HiRID: High-Resolution ICU Dataset; AUMC: Amsterdam University Medical Centers; AUROC: Area under the receiver operating characteristic curve; AUPRC: Area under the precision-recall curve.

**Table S6.** Confusion matrix counts by learning approach and site (classification threshold = 0.5).^f^

| **Approach** | **Site** | **Test set, n** | **Prevalence (%)** | **TP** | **FP** | **FN** | **TN** |
| --- | --- | --- | --- | --- | --- | --- | --- |
| **Local** | eICU-CRD | 4,521 | 28.2 | 952 | 717 | 323 | 2,529 |
|  | MIMIC-IV | 2,267 | 43.8 | 712 | 433 | 280 | 842 |
|  | UKA | 1,680 | 41.4 | 339 | 247 | 356 | 738 |
|  | HiRID | 25,423 | 17.7 | 2,740 | 3,862 | 1,769 | 17,052 |
|  | AUMC | 12,205 | 18.7 | 1,241 | 1,370 | 1,046 | 8,548 |
| **Federated** | eICU-CRD | 4,521 | 28.2 | 580 | 340 | 695 | 2,906 |
|  | MIMIC-IV | 2,267 | 43.8 | 684 | 526 | 308 | 749 |
|  | UKA | 1,680 | 41.4 | 346 | 307 | 349 | 678 |
|  | HiRID | 25,423 | 17.7 | 1,825 | 1,593 | 2,684 | 19,321 |
|  | AUMC | 12,205 | 18.7 | 1,142 | 1,177 | 1,145 | 8,741 |
| **Centralized** | eICU-CRD | 4,521 | 28.2 | 1,118 | 1,346 | 157 | 1,900 |
|  | MIMIC-IV | 2,267 | 43.8 | 904 | 842 | 88 | 433 |
|  | UKA | 1,680 | 41.4 | 613 | 762 | 82 | 223 |
|  | HiRID | 25,423 | 17.7 | 2,415 | 2,790 | 2,094 | 18,124 |
|  | AUMC | 12,205 | 18.7 | 1,275 | 1,417 | 1,012 | 8,501 |
|  | Pooled | 46,096 | 21.2 | 6,325 | 7,157 | 3,433 | 29,181 |

^f^ FN: false negatives; FP: false positives; TN: true negatives; TP: true positives. Prevalence represents the percentage of positive (successful weaning) episodes in each test set.
